# Effect of Disulfide Cyclization of Ultrashort Cationic Lipopeptides on Antimicrobial Activity and Cytotoxicity

**DOI:** 10.3390/ijms21197208

**Published:** 2020-09-29

**Authors:** Damian Neubauer, Maciej Jaśkiewicz, Emilia Sikorska, Sylwia Bartoszewska, Marta Bauer, Małgorzata Kapusta, Magdalena Narajczyk, Wojciech Kamysz

**Affiliations:** 1 Department of Inorganic Chemistry, Faculty of Pharmacy, Medical University of Gdańsk, 80-416 Gdańsk, Poland; mj@gumed.edu.pl (M.J.); sylwia.bartoszewska@gumed.edu.pl (S.B.); marta.bauer@gumed.edu.pl (M.B.); wojciech.kamysz@gumed.edu.pl (W.K.); 2 Department of Organic Chemistry, Faculty of Chemistry, University of Gdańsk, Wita Stwosza 63, 80-308 Gdańsk, Poland; emilia.sikorska@ug.edu.pl; 3 Department of Plant Cytology and Embryology, Faculty of Biology, University of Gdańsk, Wita Stwosza 59, 80-308 Gdańsk, Poland; malgorzata.kapusta@ug.edu.pl; 4 Laboratory of Electron Microscopy, Faculty of Biology, University of Gdańsk, Wita Stwosza 59, 80-308 Gdańsk, Poland; magdalena.narajczyk@ug.edu.pl

**Keywords:** antifungal, antimicrobial peptides, lipopeptides, cationic lipopeptides, short lipopeptides, cyclic lipopeptides, disulfide cyclization, disulfide bridge, *Candida*

## Abstract

Ultrashort cationic lipopeptides (USCLs) are considered to be a promising class of antimicrobials with high activity against a broad-spectrum of microorganisms. However, the majority of these compounds are characterized by significant toxicity toward human cells, which hinders their potential application. To overcome those limitations, several approaches have been advanced. One of these is disulfide cyclization that has been shown to improve drug-like characteristics of peptides. In this article the effect of disulfide cyclization of the polar head of *N*-palmitoylated USCLs on in vitro biological activity has been studied. Lipopeptides used in this study consisted of three or four basic amino acids (lysine and arginine) and cystine in a cyclic peptide. In general, disulfide cyclization of the lipopeptides resulted in peptides with reduced cytotoxicity. Disulfide-cyclized USCLs exhibited improved selectivity between *Candida* sp., Gram-positive strains and normal cells in contrast to their linear counterparts. Interactions between selected USCLs and membranes were studied by molecular dynamics simulations using a coarse-grained force field. Moreover, membrane permeabilization properties and kinetics were examined. Fluorescence and transmission electron microscopy revealed damage to *Candida* cell membrane and organelles. Concluding, USCLs are strong membrane disruptors and disulfide cyclization of polar head can have a beneficial effect on its in vitro selectivity between *Candida* sp. and normal human cells.

## 1. Introduction

**Figure 1:**
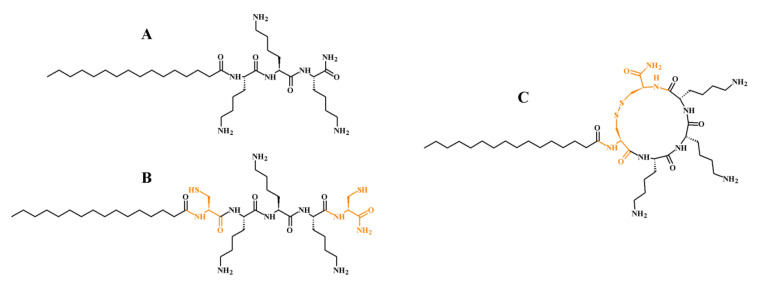
Linear lipopeptides C_16_-KKK-NH_2_ (**A**) parent molecule and its analog before (**B**) and after cyclization (**C**). Cysteine/cystine residues are colored orange.

The development of new antimicrobial agents seems to be fundamental, especially when considering dramatically increasing antimicrobial resistance [1]. This well-known fact encourages scientists to evaluate particular ideas in hope of getting closer to a solution. Nowadays, discovery of new antibiotics and chemotherapeutics has slowed down, although multidrug resistant pathogens are becoming more common. One of the promising classes of compounds is lipopeptides. Briefly, these molecules consist of a peptide linked to a fatty acid. The peptide residue is usually hydrophilic and charged, in contrast to the aliphatic lipid chain. Actually, there are only few lipopeptide antibiotics available on the market such as polymyxins, daptomycin, and echinocandins, which were originally isolated from microorganisms. Unfortunately, emergence of resistance against these antibiotics has also been reported [2,3,4]. In view of those reports, the scientific community has been forced to provide new insights into this issue and to intensify new drug development. To solve this problem, researchers have attempted to design molecules with beneficial properties to balance between toxicity and antimicrobial activity. Undoubtedly, ultrashort (up to 7 amino acid residues) cationic lipopeptides (USCLs) have been claimed to be effective antimicrobial agents [5,6,7,8]. These molecules have a detergent-like mode of action and their membrane—cation interactions partially rely on the electrostatic attraction between positively charged amino acid residues and negatively charged membrane components. The negatively charged cell surface results from the presence of lipoteichoic acid (cell wall of Gram-positive bacteria), various phospholipids in the membrane (i.e., phosphatidylserine, phosphatidylglycerol, phosphatidylinositol, and cardiolipin) as well as sialic acid (cell wall of *Candida* sp.) [9,10,11,12]. USCLs permeabilize membrane bilayers and consequently lead to cell death. Lipopeptides can exhibit, inter alia, anticancer, antibacterial, antifungal, antibiofilm, and antiadhesive activities but their application can be limited due to hemolytic potential and cytotoxicity against normal cells. Nevertheless, these properties make them perfect candidates for further studies and optimization of the structure. An appreciable antimicrobial activity of arginine- and lysine-based linear ultrashort cationic lipopeptides has been well documented [5,8,13,14,15]. A tetrapeptide containing four lysine residues and *N*-terminal hexadecenoic acid (palmitic acid, C_16_) with proven high antimicrobial activity was used as a reference lipopeptide (C_16_-KKKK-NH_2_ also known as Pal/Palm-KKKK-NH_2_) [5,16,17]. Interestingly, it has been shown that lysine-rich ultrashort *N*-palmitoylated lipopeptides can induce chemokine production in human macrophage-like THP-1 cells, this being beneficial for antibacterial therapy [18]. The mode of action of ultrashort cationic lipopeptides partially relies on electrostatic interactions with the pathogens’ membrane. It has been shown that arginine is prone to forming extensive H-bonding with membrane phospholipids thus enhancing perturbations of the pathogen membrane [19]. The lysine side chain interacts with phosphate groups of the membrane, while the arginine guanidinium group can also associate with the glycerol residue of phospholipids [20]. To evaluate the influence of arginine residue, a series of analogs with different positions of this amino acid have been synthesized. A disulfide cyclization is another approach utilized for improvement of peptides’ properties, and according to the literature, it can result in enhanced activity, selectivity, and stability [21]. Therefore, to learn how this modification can affect biological characteristics such as, antimicrobial activity against Gram-positive and Gram-negative bacteria, fungi (*Candida* sp.), and toxicity against human red blood cells (hRBCs), keratinocytes (HaCaT), and cervical cancer cells (HeLa); the set of cyclic analogs with intramolecular disulfide bridge has been synthesized. Cysteine residues were inserted at *N*- and *C*- terminal positions in the cyclic counterpart to create a loop with cystine and amino acids of the parent molecule between them. In effect, short cationic lipopeptides with arginine, cystine, lysine, hexadecenoic acid, and *C*-terminal amides were synthesized. The structures of selected lipopeptides used in this study are presented in Figure 1.

Ultrashort cationic lipopeptides (Figure 1A) and their cyclic analogs (Figure 1C) with disulfide motif were synthesized to find out how this modification can influence in vitro biological activity. Compounds with free sulfhydryl groups (Figure 1B) have not been studied herein due to their high susceptibility to oxidation [22].

## 2. Results and Discussion

### 2.1. Determination of Peptide Hydrophobicity with RP-HPLC

Lipopeptides were synthesized by solid-phase method using Fmoc chemistry. Peptides’ hydrophobicity was evaluated by analytical reversed-phase high-performance liquid chromatography (RP-HPLC). Electrospray ionization mass spectrometry (ESI MS) in positive ion mode confirmed the identity of the purified peptides. Results of HPLC and MS analyses are presented in Table 1.

**Figure 2:**
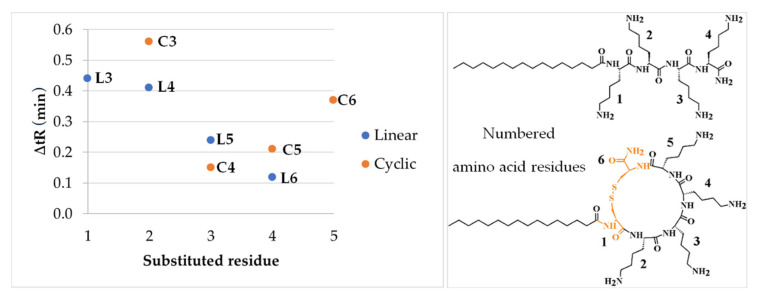
Effect of arginine position on retention behavior (left) related to unsubstituted lipopeptides (right). Cysteine/cystine residues are colored orange.

The peptides were analyzed by RP-HPLC to determine relative hydrophobicity vs. retention time of each compound. In general, more hydrophobic lipopeptides will elute later than hydrophilic ones. Using this simple rule enables comparison of hydrophobicity by reduced retention times (t’_R_) and differences in retention time between the cyclic analog and its linear parent lipopeptide (Table 1). It can be deduced that the presence of cystine moiety has led to increased hydrophobicity. All cyclic lipopeptides are more hydrophobic than their linear counterparts but the effect of disulfide cyclization on retention is different in each pair. The highest increase in retention was noticed for the shortest lipopeptides **L1** (C_16_-KKK-NH_2_) and **C1** (C_16_-CKKKC-NH_2_). The differences between lipopeptides with one arginine residue indicate that its position is crucial for retention and therefore hydrophobicity. To learn how the arginine position affects hydrophobicity, the retention time of compound **L2** (C_16_-KKKK-NH_2_) or **C2** (C_16_-CKKKKC-NH_2_) was subtracted from that of the lipopeptides with one arginine residue, linear (**L3**, **L4**, **L5**, **L6**) or cyclic (**C3**, **C4**, **C5**, **C6**), respectively. In Figure 2 the effect of arginine position on retention time is related to unsubstituted lipopeptide.

Changes in the retention (∆tR) have different patterns in the cyclic and linear lipopeptides. In the linear peptides, when arginine residue was closer to the fatty acid chain, an increase in hydrophobicity was higher (Figure 2, ΔtR of compounds **L3**, **L4**, **L5**, and **L6**). In cyclic analogs, ∆tR is the lowest for the third substituted residue, but when the fourth and fifth residues are substituted, ∆tR steeply increases. Presumably, this phenomenon can be explained in terms of the different chemical microenvironment of arginine. Residues 2 and 5 are much closer to the hydrophobic region than residues 3 and 4. In this case, it seems likely that proximity of the fatty acid chain and cysteine, as a hydrophobic part of the molecule, plays a pivotal role (Figure 2) [23]. Moreover, it has been possible to verify the additive nature of the effects of lysine substitution by arginine on hydrophobicity using the differences in ∆tR. As the substitution with arginine gives analogs with an increased retention time, it can be deduced that t’_R_ of **L7** (C_16_-RRKK-NH_2_) and **C7** (C_16_-CRRKKC-NH_2_) must be higher than that of a single substituted compound. To calculate retention time (t’_R calc._) of disubstituted lipopeptides (**L7** and **C7**), the differences in t’_R_ between unsubstituted species (**L2** or **C2**) and monosubstituted ones (**L3**, **C3**, **L4**, **C4**) were added to the retention of unsubstituted compound.

Hence,

t’_R calc. L7_ = t’_R L2_ + t’_R L3_ − t’_R L2_ + t’_R L4_ − t’_R L2_ = t’_R L3_ + t’_R L4_ − t’_R L2_ = 15.21 + 15.18 − 14.77 = 15.62 min;
t’_R exp. L7_ = 15.58 min;
t’_R calc. L7_ − t’_R exp. L7_ = 0.04 min;
t’_R calc. C7_ = t’_R C2_ + t’_R C3_ − t’_R C2_ + t’_R C4_ − t’_R C2_ = t’_R C3_ + t’_R C4_ − t’_R C2_ = 16.51 + 16.10 − 15.95 = 16.66 min;
t’_R exp. C7_ = 16.83 min;
t’_R calc. C7_ − t’_R exp. C7_ = −0.17 min.

It can be speculated that the effect of lysine substitution with arginine on hydrophobicity of linear lipopeptide is additive (t’_R calc. L7_ vs. t’_R exp. L7_). The difference between calculated and experimental retention times seems to be relatively small. The maximum coefficient of variation in this study was 0.25%, thus giving an experimental variance of ca. 0.04 min. However, in the case of cyclic lipopeptide (**C7**) a noticeable inconsistence of calculations with experiment (t’_R exp._) has been found, probably due to the consequence of interactions between neighboring arginine side-chains in disulfide-constrained peptide loop. Clusters, rings, and strings of arginine residues were found in many proteins. Interactions between guanidinium cations in arginine side-chain are known to influence the structure and stability of macromolecules [24]. Moreover, arginine was found to exhibit hydrophobic stacking, unlike lysine residues, due to energetically favorable interactions between staggered guanidinium cations [25,26]. Arginine-arginine pairing can play a crucial role in interactions of peptides and proteins such as oligomerization [27,28]. To sum up, it can be stated that it is highly likely that interactions between arginine residues are occurring in the peptides and are different in the cyclic analogs and in the linear counterpart.

### 2.2. Antimicrobial Activity of Lipopeptides

Minimum inhibitory concentrations (MICs) of lipopeptides were determined against planktonic cultures of bacteria and fungi. The results are shown in Table 2.

In the present study, all synthesized lipopeptides exhibited antimicrobial activity. Cyclization leads to compounds with similar activity against Gram-positive strains (insignificant differences). Peptides **C1** and **C5** were more active against *S. epidermidis* and *S. aureus* than their linear parent molecules. It should be noted that solubility of the compounds can be crucial for their activity. Our previous study showed that some disulfide-cyclized lipopeptides have higher antistaphylococcal activity when dissolved in aqueous solutions of acetic acid/BSA (0.01% and 0.2%, respectively) than in PBS (stock solution) [29]. Moreover, Gram-negative strains appeared to be more resistant to lipopeptides than Gram-positive bacteria and fungi, this being consistent with previous studies on lipopeptides [8,14]. Furthermore, cyclic analogs had a noticeably lower activity than the linear ones (even fourfold). The MICs of lipopeptides against Gram-negative strains ranged between 8 and 512 μg/mL. In general, *P. aeruginosa* seem to be least susceptible to USCLs. Moreover, it is noteworthy that additional lysine residue (**L2**, **C2** vs. **L1**, **C1**, respectively) gave compounds with a higher net charge (+4) and simultaneously improved antimicrobial activity against *P. aeruginosa* (contrary to *E. coli*). However, substitutions with arginine resulted in compounds with considerably lower activity against this strain. Reduced susceptibility of Gram-negative bacteria to cationic lipopeptides is mainly associated with the nature of their outer membrane. For instance, *P. aeruginosa* is equipped with a two-component regulatory system, PmrA-PmrB, that controls resistance to cationic antimicrobial peptides and lipopeptides (polymyxin B). Its activation results in incorporation of 4-aminoarabinose (l-Ara4N) into lipid A thus increasing overall charge of lipopolysaccharide (LPS) and restricting interactions between LPS and cationic lipopeptides [30,31]. Moreover, resistance to cationic antimicrobial peptides is associated with palmitoylation of lipid A in both *E. coli* and *P. aeruginosa* [32,33]. It has been documented that *E. coli* resistance to antimicrobial peptides relies partially on the outer membrane protease OmpT [34,35]. Moreover, it has been hypothesized that USCLs can form aggregates reducing their antimicrobial activity due to restrained translocation through LPS. This phenomenon is associated with the length of the fatty acid chain and composition of amino acids [36]. Both the polar head group and the fatty acid chain have a great impact on lipopeptide self-assembly. The structure and length of the fatty acid chain and amino acid composition of USCLs can affect both the oligomerization process (in solution or bound to cell) and biological activity [16,37,38,39,40].

In the present study, the lipopeptides were highly active against *Candida* strains. It has been shown that in some lipopeptides, arginine residues induced a stronger binding to the fungal model membrane than their lysine counterparts did [41]. In this study the effect of substitution of lysine with arginine residue(s) (lipopeptides **L3-L7** and **C3-C7**) on antifungal activity was position-dependent (linear and cyclic USCLs as compared to **L2** and **C2**, respectively). It seems likely that a second amino acid residue is optimal for this substitution (**L4**, **C4** vs. **L2**, **C2**) due to beneficial effect in antifungal activity in both the linear and cyclic USCLs (Table 2). Antifungal activity of the cyclic lipopeptides was usually substantially higher than that of the linear molecules (up to fourfold). Interestingly, all compounds were highly active against *C. lipolytica* and *C. tropicalis* (1–16 μg/mL). Furthermore, antifungal activity against *C. albicans* and *C. glabrata* of the linear lipopeptides (8–64 μg/mL) was reinforced upon disulfide cyclization (2–16 μg/mL). In the *Candida* strains there are two transporter families capable of transportation of peptides into the cell. Peptide transporters (PTR) are able to carry small peptides consisting of 2–3 amino acid residues into the cell. Oligopeptide transporters (OPT) are engaged in uptake of longer peptides. Some antifungal peptides need to be transported into yeast cells to induce the antifungal effect (e.g., histatin 5, Hst 5). It has been demonstrated that cysteine-rich human β-defensins require Ssa1/2 cell wall heat shock proteins for inducing antifungal activity. The same proteins are initially binding histatin 5 [42]. In fact, peptide transport deficiency can result in the resistance to some antifungals [43]. Another study revealed that polycationic Hst 5 (net charge +12) is transported through yeast polyamine transporters, Dur3p and Dur31p, and this process is energy-dependent [44]. It was shown that intracellular accumulation of cationic arginine-rich salmon protamine is required for fungicidal activity and involves cell energy. Moreover, disulfide cyclization of protamine leads to a cyclic analog with a five-fold higher anticandidal activity [45]. A hypothesis claims that disulfide cyclized USCLs can be transported into yeast cells through a transporter (OPT or a polyamine) and degrade cell membrane and its interior to cause cell death.

When a peptide is cyclic its antifungal activity is usually remarkably higher than that of the antibacterial activity as compared that of the linear parent molecules. The differences in antifungal and antibacterial (Gram-negative) activities between the cyclic and linear lipopeptides are statistically significant (*p* < 0.05). Makovitzki et al. have reported a high antimicrobial activity of C_16_-KKkK-NH_2_ (originally written as C_16_-KKKK-NH_2_; k/K—d-enantiomer) against *P. aeruginosa*, *E. coli*, and *S. aureus* (3–6.25 μM; approx. 2.3–4.8 μg/mL) being similar to that of lipopeptide **3**. However, different reference strains of *S. aureus* and *P. aeruginosa*, distinct culture media (LB) and the d-enantiomer of lysine were used, this explaining inconsistence with our results, especially those concerning Gram-negative strains (16–32 μg/mL) [5]. Again, similar results were obtained with *C. albicans* (ATCC 10231). The determined MICs were 25 μM (approx. 19.2 μg/mL) and 32 μg/mL by Makovitzki et al. and in this study, respectively. Greber et al. presented results of antimicrobial activity of C_16_-KKK-NH_2_ (**L1**) and C_16_-KKKK-NH_2_ (**L2**) against bacterial and fungal strains which only partially agree with those of this study [17]. However, the main difference in this study is related to *E. coli*, *P. aeruginosa*, and *C. albicans* strains, whose MICs are even three-fold higher for bacteria and two-fold lower for fungi. The discrepancies in antifungal activity can be explained in terms of different culture media applied for susceptibility testing. It has been well documented, also in our previous article, that the medium and cultivating conditions are critical for the determined values [29]. Different experimental conditions (initial inoculum of bacteria) can account for the observed divergence.

### 2.3. Antibiofilm Activity

Biofilms can be defined as complex conglomerates of microorganisms characterized by a significant resistance toward antibiotics. It has been reported that biofilms can be 1000-fold more resistant to antibiotics compared to that of planktonic cultures. Moreover, severe infections are often associated with biofilm formation and therefore, it seems desirable to develop new effective treatment options against this structure [46]. In view of the above, we decided to test lipopeptides in antibiofilm assay. The results of minimum biofilm eradication concentrations (MBECs) are presented in Table 3.

As a result, few cyclic lipopeptides were characterized by identical MIC and MBEC values against some Gram-negative strains (compounds **C1**, **C2**, **C5**, and **C7**) in contrast to the linear ones. Furthermore, cyclic compounds **C2**, **C4**, and **C7** had identical antibiofilm activity against bacteria as did their parent molecules. Generally, cyclic lipopeptides appeared to have even twice as high antibiofilm activity against *Candida* strains than linear lipopeptides except those with net charge +3 (**L1**, **C1**; equal MBEC values). In another study, lipopeptides C_16_-KK-NH_2_ and C_16_-RR-NH_2_ exhibited high antimicrobial activity (MIC between 4 and 32 μg/mL) and substantially lower antibiofilm potential (MBEC between 32 and >512 μg/mL) against bacteria (*S. aureus*, *S. epidermidis*, *E. coli*, *P. aeruginosa*). This general observation is comparable with that of this study [47]. Presumably, the increasing net charge improves antibiofilm activity of lipopeptides. For instance, our previous report indicated that cyclic lipopeptides exhibited higher antibiofilm activity against *Staphylococcus* strains (both the reference and clinical ones) in comparison to that of the linear molecules, but only when the compounds were dissolved in AcOH/BSA (0.01% and 0.2%, respectively) solution [29]. As the biofilm formation starts when microbial cells adhere to a surface, in terms of prophylaxis, it is desirable to protect materials from pathogen colonization, especially when taking into account its high resistance to antimicrobials. It is well documented that biosurfactants, as well as the linear and cyclic lipopeptides with palmitic acid, may prevent biofilm formation [48,49,50]. For this reason, biofilm-inhibiting properties of lipopeptides were also examined and the minimum biofilm inhibitory concentrations (MBICs) were determined. The results of MBICs are presented in Table 4.

In general, the cyclic lipopeptides tested in this study were mostly characterized by either higher or identical biofilm-inhibiting activities as that of their linear counterparts. However, few exceptions (against *E. coli*: **C1**, **C3**, **C6**, **C7**; *P. aeruginosa*: **C1**, **C7**; *C. tropicalis*: **C1**) have been found. After all, the test lipopeptides exhibited promising biofilm-inhibitory activity. With *Candida* strains, cyclic lipopeptides (mean MBIC 7.7–18.3 μg/mL) were more effective than the linear ones (13.7–86.9 μg/mL) (*p*-value; 0.003–0.097). Overall, MBICs were below MBECs (even four-fold); however, with the linear lipopeptides, the MBIC values were equal to those of MBECs (**L1**, **L3** against *S. aureus*, and **L6**, **L7** against *C. lipolytica*). For these compounds, biofilm-inhibitory properties can be the result of anti-adhesive activity based on their interactions with the microbial surface, biomaterial surface or surrounding medium [51]. Such characteristics indicate how important taking into account the material of microtiter plates while planning the microbiological assays is. In this study, polystyrene (PS) untreated (unmodified) plates were used, because ionic interactions are seemingly one of the key factors of microbial adhesion [52]. Unmodified PS is hydrophobic, and its surface can be modified on account of hydrophobic interactions between biomaterial surface and the fatty acid chain. For instance, it is well documented that surface charge on PS (either negative or positive) significantly reduces *P. aeruginosa* adhesion [53]. This finding is consistent with those of the present study for as much as the positively charged lipopeptides dissolved in the medium reduced biofilm formation of those bacteria. Similarly, in our previous article on USCLs, the MBEC and MBIC values were usually higher than the determined MICs. Moreover, the biofilm formed on contact lenses was more resistant to USCLs than that formed on polystyrene [54]. Presumably, one of the mechanisms is based on competition between negatively charged surface of a material (contact lenses; methacrylic acid of etafilcon A) and negatively charged bacterial membrane (binding of the peptide to biomaterial) for positively charged USCLs. In effect, the number of USCLs that are able to effectively disrupt pathogenic cells is reduced [55,56]. To sum up, it can be stated that one of the key factors influencing biofilm resistance to USCLs is the type of material on which it is formed.

### 2.4. MTT and Hemolysis Assay

The results of hemolytic activity and cytotoxicity of lipopeptides and selectivity indexes (SI) are presented in Table 5.

The result of cytotoxicity assays revealed that all linear lipopeptides were more cytotoxic than their cyclic counterparts (HaCaT and HeLa). However, an identical hemolysis pattern is consistent only with peptides **L4**, **C4**, **L7**, and **C7**. A common feature of these compounds is the presence of arginine residue in the second and third position, which differentiates them from the rest of compounds used in this study. Overall effect of disulfide cyclization on hemolysis seems to be dependent on arginine(s) position. In general, disulfide cyclization of lipopeptides results in the reduced cytotoxicity and enhanced hemolysis (except **C4** and **C7**). The differences in cytotoxicity against cell lines (HaCaT and HeLa) between groups of linear and cyclic lipopeptides are statistically significant (*p* < 0.05) in contrast to hemolysis. The toxicity of C_16_-KKK-NH_2_ (**L1**) against keratinocytes follows the same order of magnitude as already reported in the literature (3.2 μg/mL) [13]. It has been shown that antimicrobial activity and toxicity depend on peptide hydrophobicity and the net charge. Longer fatty acid chain leads to more active and toxic compounds, but there is an optimal chain length (hydrophobicity) required to effectively disrupt pathogenic membranes but keep the toxicity low [17,57].

**Figure 3:**
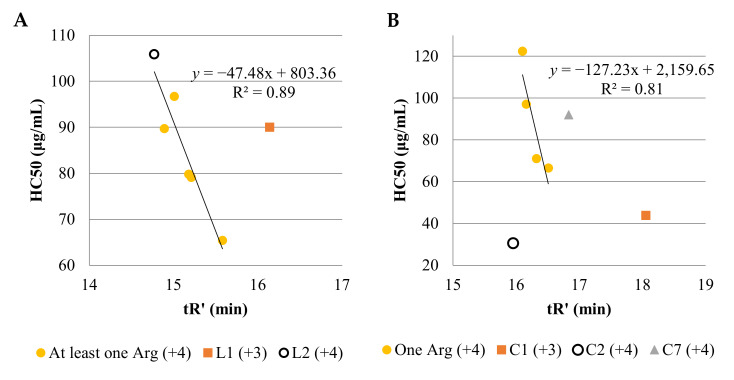
Hemolysis of—linear (**A**) and cyclic (**B**) USCLs vs. adjusted retention time. (**A**) At least one Arg (compounds **L3**, **L4**, **L5**, **L6**, **L7**)—linear correlation; (**B**) one Arg (compounds **C3**, **C4**, **C5**, **C6**)—linear correlation.

In this study, hemolysis of linear (**L1** and **L2**) and cyclic (**C1** and **C2**) USCLs with lysine residues were comparable (Table 5 and Figure 3). Interestingly, all linear lipopeptides with net charge +4 and arginine residues were more hemolytic than the four-lysine linear counterpart (**3**). In contrast to cyclic lipopeptides, all lipopeptides with an arginine residue were less hemolytic than **C2** (higher HC50 despite elevated hydrophobicity). There is a linear correlation between HC50 and hydrophobicity of linear USCLs with net charge +4 (*R*^2^ = 0.89). Similarly, HC50 of cyclic USCLs with one arginine residue (net charge +4) is linearly correlated with peptide hydrophobicity (*R*^2^ = 0.81). Cyclic lipopeptides with four lysine residues (**C2**) and two arginine residues (**C7**) do not follow the trend (Figure 3B). At the same time, the cyclic lipopeptide with two arginine residues (**C7**) exhibited unexpectedly low hemolysis especially when its highest hydrophobicity among cyclic lipopeptides with +4 net charge is taken into account. These results indicate that not only hydrophobicity, but also secondary structure, arginine position, and the number of arginine residues in cyclic lipopeptides are important for their hemolytic properties. Again, with linear lipopeptides and the highest hydrophobicity of compound **L1**, the relatively low hemolysis can be surprising. Considering the importance of peptide net charge for its biological activity, it can be presumed that low hemolysis of **L1** is due to the presence of one basic amino acid residue less (net charge +3) than in compounds with net charge +4. This finding is consistent with the literature where USCLs with greater positive charge were more toxic to human erythrocytes [17]. Similar reasoning refers to cyclic lipopeptides where compound **C1** (net charge +3) is the most hydrophobic and exhibited lower hemolysis than the most hydrophilic cyclic lipopeptide (**C2**). In other studies, USCLs with an *N*-terminal hexadecenoic acid residue and three or four ornithine residues (C_16_-Orn-Orn-Orn-NH_2_, C_16_-Orn-Orn-Orn-Orn-NH_2_), having in the side chain one methylene group less than lysine, exhibited markedly lower hemolysis than lysine counterparts **L1** and **L2**. At the same time their antimicrobial activity was comparable to those of **L1** and **L2** [58]. Indeed, exchange of lysine to ornithine can be expected to result in cyclic USCLs with elevated selectivity between hRBC and pathogens. Furthermore, all of the tested lipopeptides are substantially toxic to cancer cells—HeLa (3.0–38.8 μg/mL). Similar analysis of cytotoxicity (HaCaT and HeLa) of linear and cyclic USCLs vs. adjusted retention time was performed (Figures S1 and S2). Interestingly, cytotoxicity of compounds **C2**–**C6** to HeLa cells decreases with increasing peptide hydrophobicity (*R*^2^ = 0.88). No other correlations were observed. To assess the distinction in activity against cancer and healthy cells, the selectivity index was calculated as IC50_HaCaT_/IC50_HeLa_ (Table 5). The highest selectivity indexes (2.8 and 2.7) were found for the linear lipopeptides with one or two arginine residues at first or first and second position (**L3** and **L7**, respectively). In general, linear lipopeptides seem to be more cytotoxic to HeLa over normal cells of HaCaT than cyclic ones (mean SI_HaCaT/HeLa_ of 1.8 vs. 1.1; *p*-value 0.064). Moreover, selectivity of USCLs to pathogens (MIC) over human normal cells was estimated. Selectivity indexes were calculated as IC50_HaCaT_/MIC (SI_HaCaT_) or HC50/MIC (SI_hRBCs_). The values of SIs are attached as a Supplementary Materials (Table S1). In Table 6, arithmetic means of selectivity indexes in groups of linear and cyclic analogs are presented.

The highest SI_hRBCs_ was recorded for cyclic lipopeptides and *Candida* sp. (SI = 122.4). On the other hand, the lowest values were calculated for cyclic compounds and Gram-negative strains where hemolysis overweighed antimicrobial activity (*SI* = 0.1299). A similar spectrum of SI_hRBCs_ was observed for the linear and cyclic lipopeptides vs. Gram-positive bacteria (insignificant differences, Table 6). It is evident that cyclic molecules exhibited enhanced selectivity between fungi and HaCaT cell lines and between *S. epidermidis* and keratinocytes in contrast to those of linear counterparts. Considering antimicrobial activity against Gram-negative strains, the vast majority of compounds were nonselective. Those results are in agreement with the literature. It has been shown that cationic linear lipopeptides (net charge +2 and +3) with a hexadecanoic acid (C16) residue exhibited no selectivity between pathogens and keratinocytes (HaCaT) [13]. However, selectivity of USCL with four arginine (net charge +4) residues and hexadecanoic acid between hRBCs and bacteria/fungi based on previously determined activities is comparable to those estimated for linear USCLs used in this study [37]. In other studies, USCLs with arginine residues and shorter fatty acid chains (C14 and less) exhibited good selectivity between hRBCs, HaCaT cell line, and pathogens including bacterial strains and *C. albicans* [8,14].

### 2.5. Membrane Permeabilization

**Figure 4:**
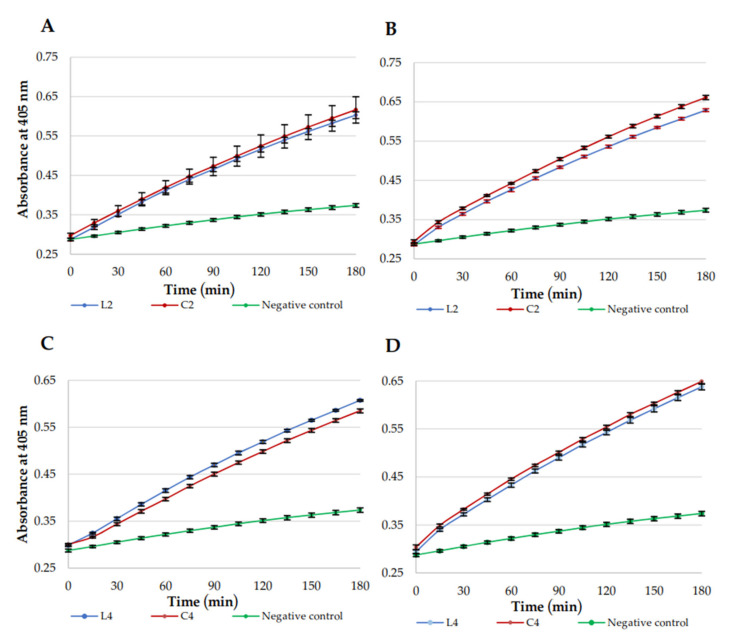
Outer membrane (OM) permeabilization kinetics. (**A**) **L2** and **C2**, 4 μg/mL; (**B**) **L2** and **C2**, 8 μg/mL; (**C**) **L4** and **C4**, 4 μg/mL; and (**D**) **L4** and **C4**, 8 μg/mL.

**Figure 5:**
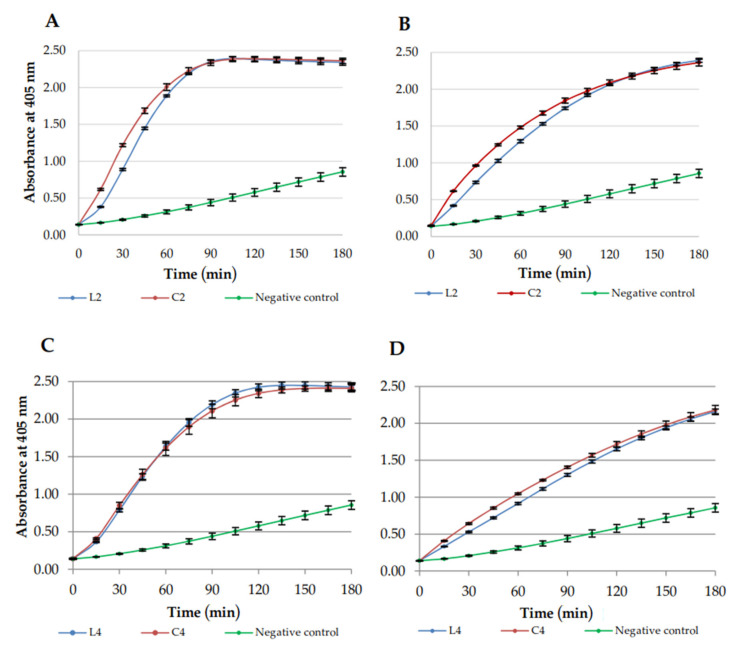
Inner membrane (IM) permeabilization kinetics. (**A**) **L2** and **C2**, 4 μg/mL; (**B**) **L2** and **C2**, 8 μg/mL; (**C**) **L4** and **C4**, 4 μg/mL; and (**D**) **L4** and **C4**, 8 μg/mL.

Permeabilization of *E. coli* ML-35 membranes was studied to compare membrane disruption caused by linear lipopeptides and their cyclic analogs. USCLs selected for this study included (**L2**) C_16_-KKKK-NH_2_, (**C2**) C_16_-CKKKKC-NH_2_ as reference lipopeptides and (**C4**) C_16_-CKRKKC-NH_2_ and linear counterpart (**L4**), owing to high selectivity indexes of the cyclic lipopeptide (highest SI_hRBCs_ = 122.40, SI_HaCaT_ = 33.80, Table S1). The MICs against *E. coli* ML-35 of selected lipopeptides were determined (linear and cyclic lipopeptides, 4 and 8 μg/mL, respectively). The outer membrane (OM) and inner membrane (IM) permeabilization studies were performed at MIC (Figure 4 and Figure 5, respectively). OM permeability studies are based on periplasmic β-lactamase action on CENTA to produce a yellow product. IM permeability studies are based on the action of cytoplasmic β-galactosidase on ONPG to produce chromophore ortho-nitrophenol (ONP). The β-galactosidase constitutive, lactose-permease deficient *E. coli* ML-35 strain was used. Accessibility of enzymes depends on OM and IM integrity. A PBS solution with ONPG/CENTA was used as a negative control.

In OM assay, cyclic and linear lipopeptides were similarly effective at both 4 and 8 μg/mL (Figure 4). However, permeabilization of IM was faster for **C2** than for linear **L2** at both concentrations (Figure 5; higher slope of a line). Similary **C4** disrupts IM more rapidly than **L4,** however, only at 8 μg/mL. USCLs achieved maximal absorbance (curve exhibiting plateau) in IM assay only at lower concentration (4 μg/mL) but no such effect was observed at 8 μg/mL. Hypothetically, this can be due to self-assembly and formation of aggregates at higher concentrations that can reduce antimicrobial activity and affect interactions with membranes. IM permeabilization curves of **L4** and **C4** at 4 μg/mL (Figure 5C) achieved plateau at approximately 120 min, whereas for **L2** and **C2** at the same concentration (Figure 5A) it was spotted at approximately 90 min. It seems that USCLs with an arginine residue (**L4**, **C4**) permeabilize IM a little slower than those lysine-based counterparts (**L2**, **C2**).

### 2.6. Membrane Depolarization properties

**Figure 6:**
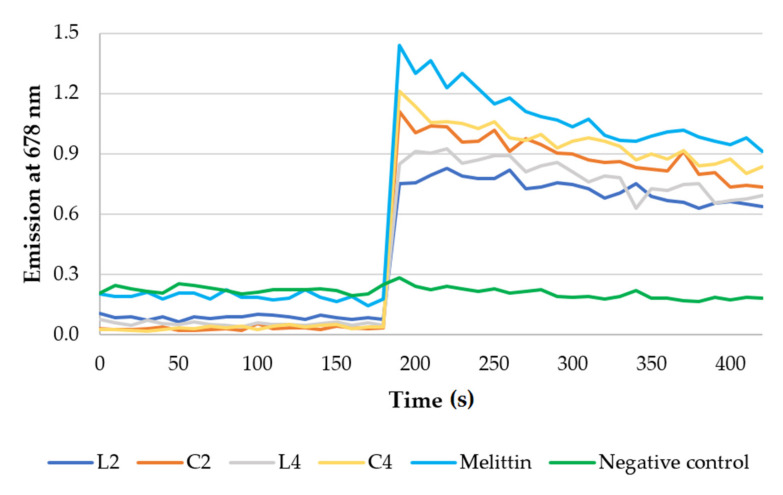
Results of fluorescence measurements of membrane potential-sensitive probe—*C. albicans*.

Membrane depolarization activities of selected lipopeptides (**L2**, **C2**, **L4**, **C4**) were evaluated using DiSC_3_(5) membrane potential-depended dye. *C. albicans*, *E. coli*, and *S. aureus* were used in this assay. Results against *C. albicans* are presented in Figure 6, while for bacteria strains are attached as Supplementary Materials (Figures S3 and S4). Melittin was used as a popular membrane disrupting peptide [59]. The concentration of melittin was 64 μg/mL.

A HEPES solution with glucose was used as a negative control. The released dye supports the hypothesis that the cell membrane is a target of synthesized lipopeptides. The results clearly indicate that the lipopeptides disrupt membranes of all pathogens used in this study. Lipopeptide C_16_-KKKK-NH_2_ (**L2**) and lipopeptides with arginine residues and hexadecanoic acid (C16) have been shown to interact with anionic lipid bilayers [16,60]. Depolarization of bacterial and fungal membranes (DiSC3(5)) was previously reported for cationic lipopeptides (net charge +4) [60].

### 2.7. Coarse-Grained Molecular Dynamics

**Figure 7:**
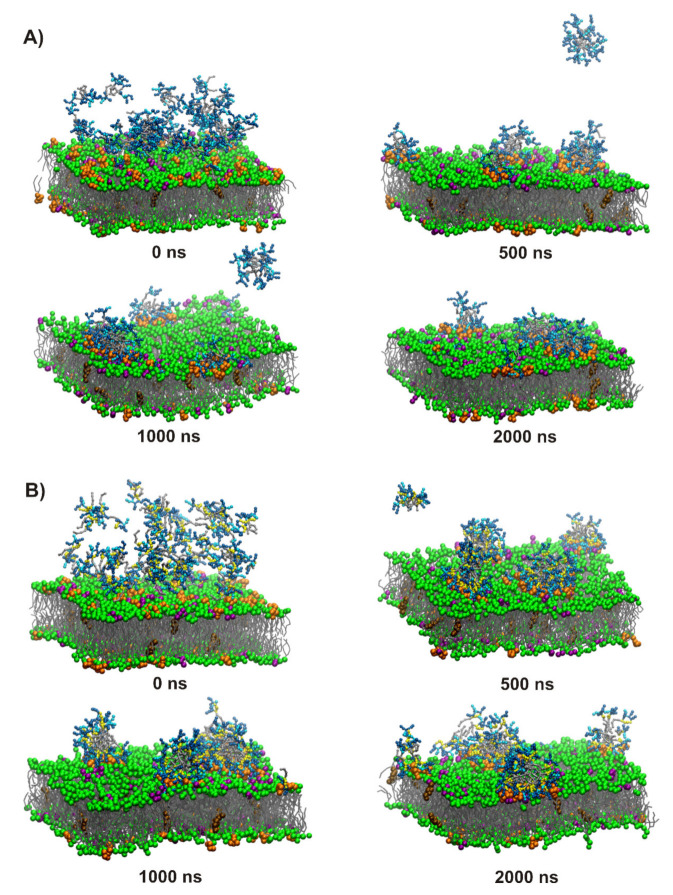
Snapshots from the POPC:POPE:POPS:POPI:ERGO (fungal membrane) binding simulations for C_16_-KRKK-NH_2_ (**A**) and C_16_-CKRKKC-NH_2_ (**B**). Fatty acid tails are colored silver, while lysines, arginines, and cysteines are blue, cyan, and yellow, respectively. Lipid tails are gray, while lipid head groups are purple for POPG, green for POPC and POPE, and orange for POPI. Ergosterol (brown) is immersed in the hydrophobic part of the membrane.

**Figure 8:**
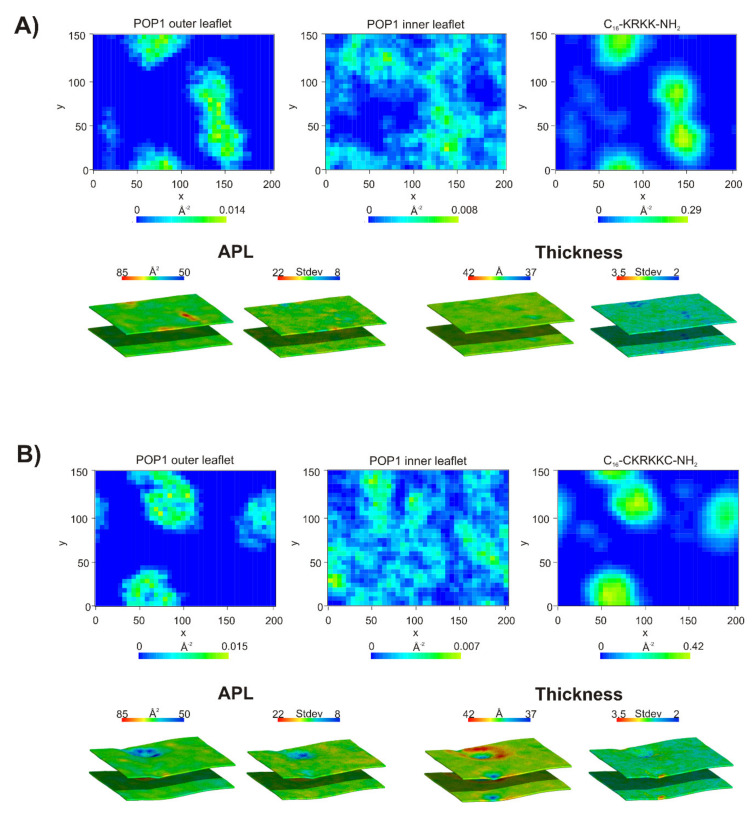
2D density map of the lipopeptides and POPI lipids in the outer and inner leaflets of the POPC:POPE:POPS:POPI:ERGO membrane (a grid spacing was set to 5 Å), local area per lipid (APL) and standard deviations of the local membrane area per lipid (APL) and local thickness of the bilayer and standard deviations of the local thickness of the bilayer averaged over the last 100 ns of a total of 2 μs CG MD (coarse-grained molecular dynamics) simulations of C_16_-KRKK-NH_2_ (**A**) and C_16_-CKRKKC-NH_2_ (**B**). Phosphate beads of the lipid headgroups were considered for calculations.

Coarse-grained molecular dynamics (CG MD) simulations were performed to visualize binding of the selected linear lipopeptides (**L2**, C_16_-KKKK-NH_2_; **L4**, C_16_-KRKK-NH_2_) and their cyclic counterparts (**C2**, C_16_-CKKKKC-NH_2_; **C4**, C_16_-CKRKKC-NH_2_) to bacterial and fungal membranes. The results showed that replacement of Lys by Arg had no significant effect on interactions of the lipopeptides with bacterial and fungal membranes in the coarse-grained model. In each system studied, two competitive processes were noticed, during the first nanoseconds of simulations, in other words, insertion of the lipopeptides into membrane matrix and self-assembly into micelles, which were attracted by the membrane surface in subsequent steps (Figure 7 and Figures S5, S7, and S9). Due to the periodic boundary conditions and crowding of the lipopeptide molecules in the initial steps of simulations, some of the molecules were free to bind with the inner leaflet of the membrane. They were removed from the system to mimic the physiological conditions, where the peptides attach only to the outer leaflet of the membrane and the simulations were continued with a reduced number of lipopeptide molecules and corrected counterions numbered. In the further steps, surface-bound lipopeptides micelles were either inserted and gradually dispersed within the outer leaflet of the membrane or remained attached to the membrane surface, with the other option being more representative of the fungal membrane. Nevertheless, even the surface-bound lipopeptide micelles affected the membrane through a distinct enrichment of POPG and POPI lipids at the binding site with the membrane surface resulting in POPG and POPI-rich domains in bacterial and fungal membrane, respectively (Figure 8 and Figures S6, S8, and S10). Formation of lipid domains resulted in nonuniform distribution of the lipids with different size headgroups and changed the local membrane thickness. The average thickness of both types of membranes is ~39.5 Å, this being compatible with that of the previous study [61]. However, in the space directly beneath the attached lipopeptide micelles, a distinct reduction in the membrane thickness and local area per lipid (APL) was noticed. Interestingly, in the case of (**C4**) C_16_-CKRKKC-NH_2_ bound to the fungal membrane (Figure 8B), a characteristic ring was formed at the binding site, in the middle of which the membrane was clearly thinner than on the outside. This indicated that (**C4**) C_16_-CKRKKC-NH_2_ induced an extensive increase in membrane permeability, which is consistent with its impressive antifungal activity.

### 2.8. Visualization of Candida albicans Cells Treated with Selected Lipopeptides by Fluorescence Microscopy and Transmission Electron Microscopy

**Figure 9:**
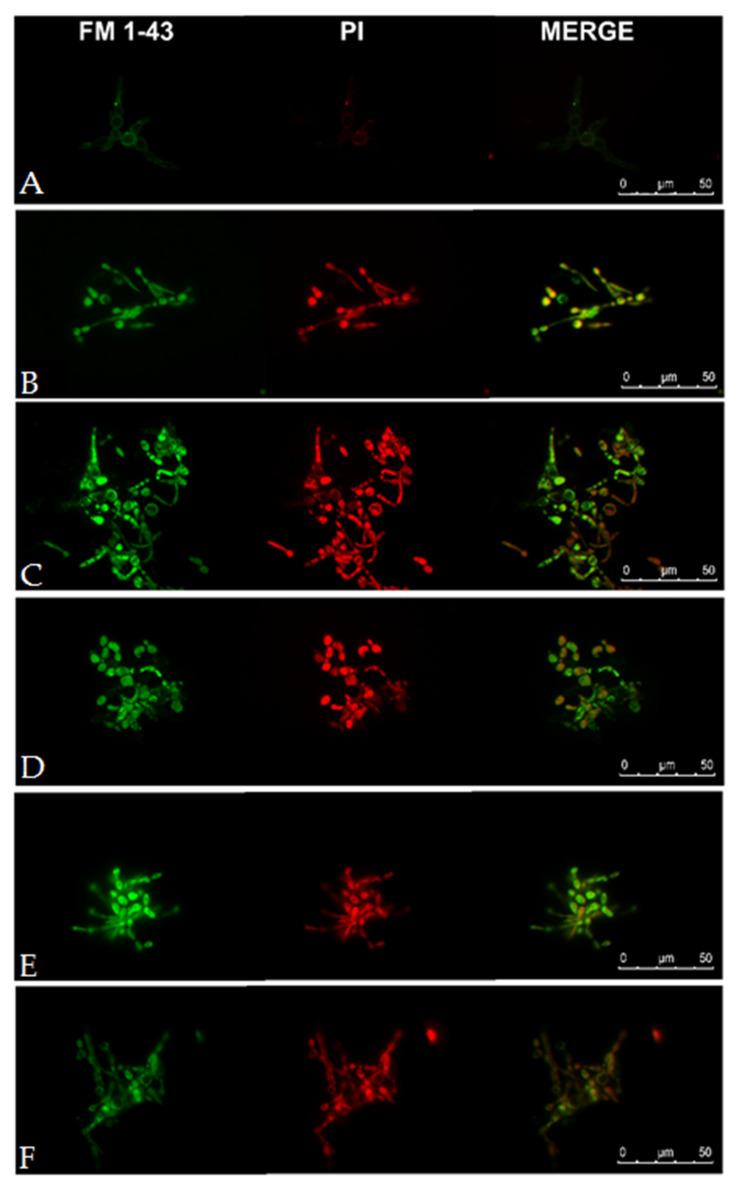
Fluorescence microscopy images of *C. albicans* ATCC 10231 cells. (**A**)—no additive, negative-control; (**B**)—melittin as positive control; (**C**)—lipopeptide **L2**; (**D**) 4—lipopeptide **C2**; (**E**)—lipopeptide **L4**; and (**F**)—lipopeptide **C4**.

As the disulfide-cyclized lipopeptide analogs exhibited exceptionally high activity against *Candida* strains, fluorescence microscopy (FM) and transmission electron microscopy (TEM) were applied to visualize perturbations of *Candida* cells caused by selected lipopeptides (**L2**, **C2**, **L4**, **C4**). Melittin was used as a popular membrane disrupting peptide (positive control). Fluorescence microscopy images are displayed in Figure 9. Dyes used in this study were *N*-(3-triethylammoniumpropyl)-4-(4-(dibutylamino) styryl) pyridinium dibromide (FM 1-43) and propidium iodide (PI).

FM 1-43 is an amphiphilic styryl green fluorescent dye that can be used to stain plasma membranes and organelle membranes in fungal cells [62]. PI needs to bind to double-stranded DNA to emit red fluorescence and cannot cross intact plasma membranes. Negative control reveals a faint green fluorescence of FM 1-43 integrated with plasma membrane and absence of red fluorescence (PI) inside the fungal cell. FM 1-43 dye can stain internal membranes, especially mitochondria ca. 30 min after dye addition [63]. Observations were made immediately after peptide addition to eliminate natural dye uptake (endocytosis). Lipopeptides, similar to melittin, permeabilize cell membranes facilitating dye penetration and binding with internal membranes (green fluorescence). Red fluorescence from PI confirmed permeabilization of cell membrane of yeast treated with both lipopeptides (**L2**, **C2**, **L4**, **C4**) and melittin.

**Figure 10:**
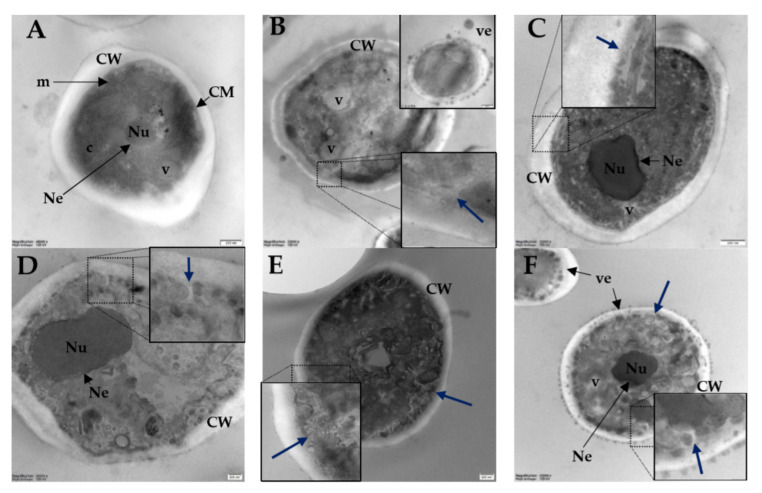
Transmission electron microscopy (TEM) images of *C. albicans* ATCC 10231 cells. (**A**)—no additive, negative-control; (**B**)—melittin as positive control; (**C**)—lipopeptide **L2**; (**D**)—lipopeptide **C2**; (**E**)—lipopeptide **L4**; and (**F**)—lipopeptide **C4**. CM—cell membrane, CW—cell wall, c—cytoplasm, m—mitochondrion, Nu—nucleus, Ne—nuclear envelope, v—vacuole, ve—vesicles.

Transmission electron microscopy images are presented in Figure 10.

In TEM micrographs of untreated cells (Figure 10A) a typical morphology is seen. Normal cells have a regular thick cell wall, continuous cell membrane, a noticeable nucleus surrounded by intact nuclear envelope, and numerous vacuoles and mitochondria in the cytoplasm. Cells treated with melittin and lipopeptides have membranes detached from the cell wall with plasma membrane invaginations (blue arrows). Moreover, all compounds caused multiple small lobes of vacuoles. In the micrographs of cells treated with melittin and lipopeptide **C4** numerous vesicles are visible inside and outside the cell. These bleb-like structures over the *Candida albicans* cells treated with melittin were seen previously using scanning electron microscopy [64]. Similar bleb-like structures occurred in Gram-negative bacteria treated with polymyxin B and colistin, cationic cyclic lipopeptide antibiotics [65,66]. Undoubtedly, all peptides caused disintegration of cytoplasm and organelles. None of the peptides caused damage to the cell wall. These results are consistent with a previous study on lipopeptides with lysine residues and palmitic acid attached to the *N*-terminus, where damage to the membrane was reported [40,67].

## 3. Materials and Methods

### 3.1. Peptide Synthesis

Peptides were synthesized manually by solid-phase Fmoc/tBu methodology. Polystyrene resin modified by Rink Amide linker was used as the solid support. Deprotection of the Fmoc group was performed with a 20% (*v*/*v*) piperidine solution in DMF for 15 min. Acylation was conducted with a mixture of DIC:OxymaPure:Fmoc-AA-OH (mole ratio 1:1:1) dissolved in DMF:DCM (1:1, *v*/*v*) in fourfold excess based on the resin for 1.5 h. After deprotection and coupling reactions, the resin was rinsed with DMF and DCM and subsequently the chloranil test was carried out. The peptides were cleaved from the resin using one of the mixtures; (A) TFA, EDT, TIS, and water (92.5:2.5:2.5:2.5 *v*/*v*/*v*/*v*); (B) TFA, TIS, and water (95:2.5:2.5 *v*/*v*/*v*). Mixture (A) was used with peptides containing a cysteine residue, whereas mixture (B) for the remaining peptides. Cleavage was accomplished within 1.5 h under stirring. Then the peptides were precipitated with cooled diethyl ether and lyophilized. The crude peptide with cysteine was dissolved in 20% (*v*/*v*) acetic acid solution (0.5 g/L) and oxidized with iodine to obtain the peptide with intramolecular disulfide bridge. The peptides were purified by RP-HPLC. Pure fractions (>95%, HPLC) were collected and lyophilized. The identity of all compounds was confirmed by mass spectrometry (ESI–MS). Melittin (GIGAVLKVLTTGLPALISWIKRKRQQ-NH_2_) used in this study was also synthesized on solid-support as first described. It was cleaved from the resin for 1.5 h using a TFA, TIS, phenol, and water (92.5:2.5:2.5:2.5, *v*/*v*/*v*/*v*) mixture.

### 3.2. Determination of Peptide Hydrophobicity with RP-HPLC

To determine peptide hydrophobicity, a Waters Alliance e2695 system with a Waters 2998 PDA Detector (software-Empower 3, Waters, Milford, MA, USA) was used. All analyses were carried out on a Waters X-Bridge Shield RP-18 column (3.0 × 100 mm, 3.5 μm particle size, 130 Å pore size). The Shield Technology column with embedded polar groups was used to minimize interactions of unreacted silanol groups with basic lipopeptides [68]. The peptides were dissolved in water (0.1% TFA, *v*/*v*) up to a concentration of 1 g/L. UV detection at 214 nm was used, and samples (10 μL) were eluted with a linear 20–65% acetonitrile gradient in deionized water over 30 min at 25.0 ± 0.1 °C. The mobile phase flow rate was 0.5 mL/min. Both eluents contained 0.1% (*v*/*v*) of TFA. Each peptide sample was analyzed in triplicate. Maximum standard deviation and coefficient of variation were 0.042 and 0.25%, respectively.

### 3.3. Antimicrobial Activity

#### 3.3.1. Cultivation of Microorganisms

The *Staphylococcus aureus* ATCC 25923, *Staphylococcus epidermidis* ATCC 14990, *Escherichia coli* ATCC 25922, *Pseudomonas aeruginosa* ATCC 9027, *Candida albicans* ATCC 10231, *Candida glabrata* ATCC 15126, *Candida lipolytica* PCM 2680-FY, and *Candida tropicalis* PCM 2709-FY strains were acquired from the Polish Collection of Microorganisms (PCM, Polish Academy of Sciences, Wrocław, Poland) and from the American Type Culture Collection (ATCC). All the strains were stored at −80 °C in Roti-Store cryo vials and before the tests were transferred into fresh Mueller–Hinton broth (MHB, Biocorp, Warsaw, Poland) for bacteria or RPMI-1640 (Sigma-Aldrich, Steinheim, Germany) for fungi and incubated for 24 h at 37 °C. Then, the cultures were seeded on Mueller–Hinton agar (BioMaxima, Lublin, Poland) or Sabouraud dextrose agar (SDA, BioMaxima, Lublin, Poland) plates, respectively, and incubated as just mentioned. These agar cultures were used for further microbiological assays. Cell densities for all assays were adjusted spectrophotometrically (Multiskan GO Microplate Spectrophotometer, Thermo Fisher Scientific, Vantaa, Finland) at 600 and 660 nm for bacteria and fungi, respectively.

#### 3.3.2. Activity against Planktonic Cultures

The MICs were determined by broth microdilution method according to the Clinical and Laboratory Standard Institute guidelines [69,70] For this purpose, initial inoculums of bacteria (5 × 10^5^ CFU/mL) in MHB and yeasts (2 × 10^3^ CFU/mL) in RPMI-1640 with 2% D-glucose were exposed to the ranging concentration of lipopeptides (0.5–256 μg/mL) and incubated at 37 °C for 18 h and 24 h, respectively. The experiments were conducted on 96-well microtiter polystyrene plates. The growth was assessed visually after incubation and the MIC was assumed as the lowest peptide concentration at which a noticeable growth of microorganisms was inhibited. All experiments were conducted in triplicate.

#### 3.3.3. Activity against Biofilm

MBECs were determined on 96-well polystyrene flat-bottom plates. For this purpose, 24 h cultures of microorganisms were diluted to obtain final density 5.0 × 10^5^ CFU/mL and 2.0 × 10^5^ CFU/mL of bacteria and fungi, respectively. Briefly, 100 µL of cell suspension was added into the test plates. After 24 h of incubation at 37 °C the wells were rinsed three times with a phosphate buffer saline (PBS, pH 7.4) to remove non-adherent cells. Subsequently, 100 μL of the test compounds in concentration range (0.5–256 μg/mL) were added to each well. After 24 h of incubation at 37 °C, 20 μL of a cell viability reagent was added (resazurin, 4 g/L; Sigma Aldrich, St. Louis, MO, USA). The MBEC was read after 1 h. The determined values were recorded as the lowest concentration at which the reduction of resazurin (from blue to pink) was lower or equal to 10 ± 0.5% as compared to the positive (100%) and negative (0%) controls. The reduction was monitored by measuring absorbance at 570 nm (reduced) and 600 nm (oxidized) using a microplate spectrophotometer (Multiskan GO Microplate Spectrophotometer, Thermo Fisher Scientific, Vantaa, Finland). All experiments were conducted in triplicate.

#### 3.3.4. Biofilm Inhibition Assay

The assay was performed to evaluate the effect of the lipopeptides in preventing biofilm formation. The preparation of inoculums was followed by a ca. 500-fold dilution of the 24 h cultures of microorganisms. Briefly, 50 μL of the compounds in the concentration range, diluted in appropriate medium, were prepared on 96-well polystyrene flat-bottom plates. Subsequently, the 50 μL of the bacterial/fungal inoculums were added to reach the same microbial density as that in the MBEC assay. After 24 h of incubation at 37 °C, the wells were rinsed three times with PBS and the fresh media with resazurin (4 g/L) were added. The MBICs were read after 1 h. All experiments were conducted in triplicate.

### 3.4. Permeabilization of E. coli ML-35 Membranes

To assess lipopeptides ability to permeabilize inner (IM) and outer (OM) membranes of Gram-negative bacteria, the *E. coli* ML-35 (ATCC 43827) strain was used. The strain produces cytoplasmic β-galactosidase, periplasmic β-lactamase, and lacks lac permease. To examine OM permeabilization, CENTA was used as a β-lactamase substrate. In effect of OM permeabilization, the enzyme can hydrolyze CENTA’s β-lactam ring. The resulting color change can be measured spectrophotometrically at 405 nm [71]. The IM permeabilization was monitored with ONPG, a chromogenic β-galactosidase substrate. The product of this reaction (4-nitrophenol) was measured spectrophotometrically at 405 nm. Lipopeptide concentrations in OM and IM permeabilization assays were MIC (4 μg/mL of **L2** and **L4,** and 8 μg/mL of **C2** and **C4**). Bacteria were incubated in LB medium for 24 h at 37 °C. Subsequently, the cells were diluted in a fresh LB medium and incubated at 37 °C up to a mid-log phase (ca. 3 h). The culture was centrifuged (3 min, 1100× *g*) and rinsed twice with a PBS. The bacteria were resuspended in PBS up to a concentration of 5 × 10^6^ CFU/mL. The final concentration of ONPG and CENTA was 1.5 mM and 0.15 mM, respectively. The ONPG/CENTA in PBS was used as negative control. Readings were taken every 15 min for 3 h at 37 °C [72,73,74,75]. Experiments were performed in triplicate on 96-well microtiter polystyrene plates.

### 3.5. Membrane Depolarization Assay

Depolarization of cell membranes was measured with 3,3′-dipropylthiacarbocyanine (diSC3(5)) as a probe. Membrane permeabilization could be assessed due to its depolarization and simultaneous release of the probe to the medium. Only the released dye can emit fluorescence to indicate membrane damage. *S. aureus* ATCC 25923, *E. coli* ATCC 25922, and *C. albicans* ATCC 10231, were grown at 37 °C up to a mid-log phase (approx. 4 h) in Mueller–Hinton (bacteria) or RPMI-1640 (fungi) broth. The cultures were centrifuged (3500 rpm, 7 min) and washed with 20 mM glucose solution in HEPES buffer (5 mM, pH 7.2). The cells were resuspended in 5 mM HEPES buffer supplemented with 20 mM glucose and 100 mM KCl (pH 7.2) to an OD 0.05 (OD600 and OD660 for bacteria and fungi, respectively). The final concentration of the dye was 0.4 μM. Fluorescence were monitored at 20 °C (λ_ex_ 620 nm and λ_em_ 678 nm) with Fluoroskan AscentFL (Thermo Fisher Scientific) fluorometer. As soon as the dye uptake attained a maximum the peptides were added at a concentration of 2 × MIC. Melittin is known as an effective membrane disruptor, and as such it was used as a positive control (2 × MIC; 64 μg/mL). A HEPES solution with glucose was used as a negative control. The measurements were run twice to ensure reproducibility.

### 3.6. Hemolysis Assay

The hemolysis assay was performed by using the method reported previously [8,60]. Fresh human red blood cells (hRBCs) with anticoagulant (EDTA) were rinsed three times with a PBS by centrifugation at 800× *g* for 10 min and diluted with PBS. The lipopeptides were serially diluted on a 96-well microtiter polystyrene plate and hRBCs were added up to a final volume of 100 μL. The peptide concentration ranged between 0.5 and 256 μg/mL and the final hRBCs concentration was 4% (*v/v*). Controls for zero hemolysis (blank) and 100% hemolysis consisted of hRBCs suspended in PBS and 1% of Triton-X 100, respectively. The plate was incubated for 1 h at 37 °C and then centrifuged (800× *g*, 10 min, 4 °C). Subsequently, the supernatant was resuspended to new microtiter plates and the absorbance at 540 nm was measured. All experiments were conducted in triplicate. HC50 was calculated using an ic50.tk tool. Protocol of the study received approval from the local Bioethics Committee at the Medical University of Gdańsk (NKBBN/262/2019, approval date: 10 June 2019).

### 3.7. MTT Assay

To assess the cytotoxicity of the test lipopeptides (IC50), the classic MTT assay on 96-well polystyrene plates was performed for human keratinocytes (HaCaT) and human cervical adenocarcinoma cell line (HeLa S3) acquired from the ATCC. The assay utilizes colorimetric determination of the cell metabolic activity and the color intensity reflects the number of live cells that can be measured spectrophotometrically. The cell line was cultured in a Dulbecco’s modified Eagle Medium (Invitrogen) supplemented with 10% fetal bovine serum (*v*/*v*), 100 units/mL of penicillin, 100 μg/mL of streptomycin, and 2 mM l-glutamine and was stored at 37 °C in a humidified 5% CO_2_ incubator. Briefly, a day after plating of 500 cells per well, a series of concentrations (0.1–200 μg/mL) of the test compounds were applied. DMSO was added to the control cells at a final concentration of 1.0% (*v*/*v*), which was related to the maximal concentration of the solvent compounds used in the experiment. After 24 h of incubation with the lipopeptides at 37 °C in a humidified 5% CO_2_ incubator, a medium containing 1 mg/mL of MTT (3-(4,5-dimethylthiazol-2-yl)-2,5-diphenyltetrazolium bromide) was added to the wells up to a final concentration of 0.5 mg/mL. Subsequently, the plates were incubated at 37 °C for 4 h. Then, the medium was removed by suction, and the formazan product was solubilized with DMSO. The background absorbance at 630 nm was subtracted from that at 570 nm for each well (Epoch, BioTek Instruments, Winooski, VT, USA). Six replicates were conducted for each concentration. All experiments were repeated at least twice and the resulting IC50 values were calculated with a GraFit 7 software (v. 7.0, Erithacus, Berkley, CA, USA).

### 3.8. Fluorescence Microscopy

Fungal cells (*Candida albicans* ATCC 10231) were incubated in RMPI-1640 overnight. The cells were then centrifuged (3500 rpm, 7 min) and resuspended in a fresh RMPI-1640 medium to obtain dense cell suspension (0.5 × 10^7^ CFU/mL). The cells were subsequently treated with USCLs and melittin at 2 × MIC. Fungi were stained with 10 μM FM 1-43 Dye (Thermo Fischer Scientific) and 2.5 μg/mL propidium iodide (PI, Sigma Aldrich) and immediately visualized using Nikon ECLIPSE E800 microscope with FITC and rhodamine filter (G-2A), respectively.

### 3.9. Transmission Electron Microscopy (TEM)

TEM was used to examine ultrastructural changes in the *Candida albicans* ATCC 10231 cells treated with selected lipopeptides (**L2**, **C2**, **L4**, **C4**) and melittin (positive control). Fungal cells (*Candida albicans* ATCC 10231) were incubated in RMPI-1640 overnight. The cells were centrifuged (3500 rpm, 7 min) and resuspended in fresh RMPI-1640 medium to obtain dense cell suspension (5.0 × 10^6^ CFU/mL). The high cell-density culture was prepared to facilitate TEM observations. Peptides’ concentrations were 2 × MIC. Samples were incubated for 1 h at room temperature. Importantly, MIC of selected peptides (**L2**, **C2**, **L4**, **C4,** and melittin) were also determined with dense inoculum of yeasts (5.0 × 10^6^ CFU/mL). After the treatment, the fungal cells were centrifuged (3500× rpm, 10 min) and washed twice with PBS. Fungal cells were fixed with 2.5% glutaraldehyde buffered at pH 6.5 with 0.1 M sodium cacodylate (Polysciences, Warrington, PA, USA) for 6 h at room temperature. Postfixation was performed with a 1% osmium tetroxide (Polysciences, Warrington, PA, USA) solution for 2 h at 4 °C. The cells were then centrifuged and resuspended in 0.1 M cacodylate buffer (pH 6.5). After re-centrifugation, cells were dehydrated with ethanol. Yeasts were embedded in Epon 812 resin (Sigma-Aldrich) at room temperature. An ultramicrotome Leica UC7 was used to prepare ultrathin sections (55 nm). Lead citrate and uranyl acetate were added as contrasting agents. The cells were visualized using a Tecnai Spirit BioTWIN microscope (FEI) at 120 kV.

### 3.10. Molecular Dynamics Simulations

Simulations were carried out in the MARTINI coarse-grained force field [76,77] implemented in the GROMACS 4.6.3 package [78]. The model of fungal membrane was built from 958 lipids consisting of 485 POPC, 288 POPE, 94 POPS, and 94 POPI (mole ratio 5:3:1:1) equally distributed between both leaflets. Additionally, the fungal membrane included 2% of ergosterol (11 molecules per each leaflet) [61]. In turn, the bacterial membrane was built from 986 lipids consisting of POPG and POPE lipids at a mole ratio of 3:1, which mimicked the Gram-positive bacterial membrane [79]. Each system was constructed using an insane.py script available on the martini website (http://cgmartini.nl/cgmartini/). One hundred molecules of each lipopeptide were placed randomly on one leaflet of the membrane. The lipid to lipopeptide ratio was ca. 10:1. The entire system was solvated and neutralized by sodium and chloride ions to keep the concentration of the free salt ions at ca. 100 mM. Simulations of lipopeptide-membrane interactions were carried out in an isothermal-isobaric (NPT) ensemble with semi-isotropic pressure of 1 bar and at a fixed temperature of 303 K. The CG MD simulations for each system were run for 2 μs. The time step of 10 fs, as suggested by Winger et al. [80], was employed during the entire NPT simulations. The temperature was held at 303 K using the Nosé–Hoover temperature coupling. The pressure was treated semi-isotropically at 1 bar using the Parinello–Rahman barostat with a coupling constant *τ_p_* = 12 ps and an isothermal compressibility of 4.5 × 10^−5^ bar^−1^. The relative dielectric constant for explicit screening was 15. Electrostatics were computed with a shift function with a coulomb cutoff of 12 Å. The shift function was used for van der Waals interactions as well, with a switch distance of 9 Å and a cutoff of 12 Å. The neighbor list was updated every 20 steps using a cutoff 14 Å. MD trajectory analysis was performed with the utilities included in the GROMACS package. Area per lipid (APL) was calculated using the GROMACS compatible analysis tool g_lomepro [81]. Visualizations were created with VMD [82].

### 3.11. Statistical Analysis

The results of antimicrobial activity (MIC, MBEC, MBIC) and cytotoxicity (HC50, IC50) studies were analyzed statistically. The peptides were divided into two equinumerous groups (*n* = 7) of linear and cyclic lipopeptides. A Shapiro–Wilk test was performed to evaluate normality in each group. When the data were normally distributed, a Levene’s test was performed to study homogeneity of variance. Differences between groups were tested by either Student’s *t*-test or Mann–Whitney U test. The Student’s *t*-test was used when data were normally distributed, and variances were homogenous. If not, the Mann–Whitney U test was used. The significance level (α) was 0.05. The analyses were performed with TIBCO Statistica 13.3.0.

## 4. Conclusions

In this article, in vitro biological activities of *N*-palmitoylated linear and cyclic lipopeptides were studied. All of the cyclic lipopeptides had a substantially high antimicrobial activity (MIC, MBEC) against *Candida* strains and low cytotoxicity against keratinocytes (HaCaT) in comparison with their linear counterparts. Generally, cyclic lipopeptides caused higher hemolysis (HC50) than did their parent molecules, but with a few exceptions. Furthermore, substitution of lysine with arginine led to analogs with a lower hemolytic activity but only in the case of cyclic lipopeptides. The most selective compound was a cyclic lipopeptide with one arginine and three lysine residues, (**C4**) C_16_-CKRKKC-NH_2_. Compounds used in this study exhibited distinct biofilm-inhibitory properties promoting them as candidates applicable in biomaterials and polymers. It has been shown that USCLs can be promising anti-biofilm and antimicrobial coatings and components of formulations with biodegradable polymers [50,83]. All in all, short cationic lipopeptides are molecules with high antimicrobial activity. Unfortunately a noticeable toxicity against human cells is a common feature of this class of compounds. In order to overcome this drawback, lipopeptides’ polar head could be simply cyclized through disulfide formation. Moreover, this approach can lead to compounds with improved antimicrobial activity and selectivity between *Candida* strains and human cells. Further studies should include analysis of the hydrophobic fragment of the presented cyclic lipopeptides. Shortening of the fatty acid chain and addition of hydrophobic *N*-terminal amino acid residue can result in more active and selective compounds [8]. Furthermore, stability to enzymatic degradation should be considered especially with respect to the disulfide bridge that can be reduced or eventually (re)oxidized or conjugated to another sulfhydryl. A number of chemical strategies can be applied to overcome this drawback, for example, thioether bond or amide bond formation, with lipidation of the *N*-terminus, side-chain amino or hydroxyl group or even cysteine, in other words, through CLipPA chemistry using a fatty acid vinyl ester [84,85,86]. Another aspect that can be crucial to antimicrobial activity of cyclic ultrashort cationic lipopeptides is ring size. It was found that the ring size of cyclized antimicrobial peptides and lipopeptides (polymyxin B, echinocandins) was an essential factor that can affect biological activity [87,88,89,90].

## Figures and Tables

**Table 1:** MS analysis, reduced retention time of lipopeptides (t’R), and calculated differences in retention time between the parent molecule and its analog (∆tR).

Peptide Code	Sequence	Av. Mass (Da)	Monoisotopic Mass (Da)	MS Analysis			Net Charge	t’_R_ (min)	∆t_R_ (min) *
				z ^a^	*m*/*z* Calc.	*m*/*z* Found			
**L1**	C_16_-KKK-NH_2_	639.97	639.54	1	640.55	640.69	+3	16.14	+1.91
				2	320.78	321.01			
				3	214.19	214.39			
**C1**	C_16_-CKKKC-NH_2_	844.24	843.54	1	844.55	844.70	+3	18.05	
				2	422.78	423.04			
				3	282.19	282.43			
**L2**	C_16_-KKKK-NH_2_	768.14	767.64	1	768.64	768.86	+4	14.77	+1.18
				2	384.83	385.18			
				3	256.89	257.25			
				4	192.92	-			
**C2**	C_16_-CKKKKC-NH_2_	972.42	971.64	1	972.65	972.75	+4	15.95	
				2	486.83	487.19			
				3	324.89	325.20			
				4	243.92	-			
**L3**	C_16_-RKKK-NH_2_	796.15	795.64	1	796.65	796.84	+4	15.21	+1.30
				2	398.83	399.14			
				3	266.22	266.59			
				4	199.92	-			
**C3**	C_16_-CRKKKC-NH_2_	1000.43	999.65	1	1000.65	1000.83	+4	16.51	
				2	500.83	501.19			
				3	334.22	334.62			
				4	250.92	-			
**L4**	C_16_-KRKK-NH_2_	796.15	795.64	1	796.65	769.95	+4	15.18	+0.92
				2	398.83	399.27			
				3	266.22	266.54			
				4	199.92	-			
**C4**	C_16_-CKRKKC-NH_2_	1000.43	999.65	1	1000.65	1000.86	+4	16.10	
				2	500.83	501.07			
				3	334.22	334.68			
				4	250.92	-			
**L5**	C_16_-KKRK-NH_2_	796.15	795.64	1	796.65	796.80	+4	15.01	+1.15
				2	398.83	399.24			
				3	266.22	266.53			
				4	199.92	-			
**C5**	C_16_-CKKRKC-NH_2_	1000.43	999.65	1	1000.65	1000.77	+4	16.16	
				2	500.83	501.11			
				3	334.22	334.45			
				4	250.92	-			
**L6**	C_16_-KKKR-NH_2_	796.15	795.64	1	796.65	796.81	+4	14.89	+1.43
				2	398.83	399.11			
				3	266.22	266.42			
				4	199.92	-			
**C6**	C_16_-CKKKRC-NH_2_	1000.43	999.65	1	1000.65	1000.80	+4	16.32	
				2	500.83	501.11			
				3	334.22	334.51			
				4	250.92	251.11			
**L7**	C_16_-RRKK-NH_2_	824.17	823.65	1	824.66	824.87	+4	15.58	+1.25
				2	412.83	413.21			
				3	275.56	276.02			
				4	206.92	-			
**C7**	C_16_-CRRKKC-NH_2_	1028.44	1027.65	1	1028.66	1028.79	+4	16.83	
				2	514.83	515.20			
				3	343.56	343.89			
				4	257.92	-			

^a^ ion charge; * ∆t_R_ = t_R_ of cyclic analog—t_R_ of parent lipopeptide.

**Table 2:** Antimicrobial activity of lipopeptides.

Peptide Code	MIC (μg/mL)							
	*S. aureus*	*S. epidermidis*	*E. coli*	*P. aeruginosa*	*C. albicans*	*C. glabrata*	*C. lipolytica*	*C. tropicalis*
**L1**	32 *	8	16	64	32	32	16	8
**C1**	8 *	4	16	256	8	4	4	4
**L2**	16	8	32	16	32	64	8	4
**C2**	32	8	64	32	4	4	2	2
**L3**	16 *	8	8	32	8	64	8	8
**C3**	8 *	8	16	512	2	8	4	2
**L4**	8	8	8	32	16	32	1	2
**C4**	16	8	32	256	2	4	1	2
**L5**	16 *	8	8	64	32	64	8	8
**C5**	8 *	4	16	256	16	8	4	4
**L6**	16 *	4	8	64	32	64	8	8
**C6**	16 *	8	32	128	4	4	2	2
**L7**	4	4	8	64	32	16	8	8
C7	8	4	16	256	16	4	4	4
L_mean_ **	15.4	6.9	12.6 ^†^	48.0 ^†^	26.3 ^†^	48.0 ^†^	8.1 ^†^	6.6 ^†^
C_mean_ **	13.7	6.3	27.4 ^†^	242.3 ^†^	7.4 ^†^	5.1 ^†^	3.0 ^†^	2.9 ^†^
*p*-value	0.633	0.701	0.030	0.015	0.009	0.002	0.025	0.025

* These analyses were performed by our group in the previous study [29]; ** arithmetic mean of Minimum inhibitory concentrations (MIC) for linear (L) and cyclic (C) lipopeptides. † *p*-values for significant differences (*p* < 0.05).

**Table 3:** Antibiofilm activity of lipopeptides.

Peptide Code	MBEC (μg/mL)							
	*S. aureus*	*S. epidermidis*	*E. coli*	*P. aeruginosa*	*C. albicans*	*C. glabrata*	*C. lipolytica*	*C. tropicalis*
**L1**	64 *	32	64	128	256	256	64	128
**C1**	64 *	32	256	256	256	256	64	128
**L2**	64	32	64	>256	256	256	64	128
**C2**	64	32	64	> 256	64	64	16	32
**L3**	64 *	32	128	256	128	128	32	32
**C3**	128 *	128	256	256	32	32	16	32
**L4**	64	32	64	>256	128	128	32	64
**C4**	64	32	64	>256	32	64	16	32
**L5**	32 *	32	64	256	256	256	64	128
**C5**	128 *	64	128	256	64	64	16	32
**L6**	32 *	32	256	256	128	128	32	128
**C6**	64 *	128	>256	>256	64	64	16	32
**L7**	128	64	256	256	64	128	16	64
**C7**	128	64	256	256	64	64	16	32
L_mean_ **	64.0	36.6	-	-	173.7 ^†^	182.9 ^†^	43.4 ^†^	96.0 ^†^
C_mean_ **	91.4	68.6	-	-	82.3 ^†^	86.9 ^†^	22.9 ^†^	45.7 ^†^
*p*-value	0.131	0.160	-	-	0.035	0.018	0.048	0.041

* These analyses were performed by our group in the previous study [29]; ** arithmetic mean of minimum biofilm eradication concentrations (MBEC) for linear (L) and cyclic (C) lipopeptides. † *p*-values for significant differences (*p* < 0.05).

**Table 4:** Biofilm inhibitory activity of lipopeptides.

Peptide Code	MBIC (μg/mL)							
	*S. aureus*	*S. epidermidis*	*E. coli*	*P. aeruginosa*	*C. albicans*	*C. glabrata*	*C. lipolytica*	*C. tropicalis*
**L1**	32	16	32	32	128	128	32	16
**C1**	16	16	64	128	32	32	16	32
**L2**	32	16	32	64	128	128	32	16
**C2**	16	4	32	64	16	32	8	8
**L3**	32	16	32	64	64	64	16	16
**C3**	16	8	64	64	8	8	2	4
**L4**	16	8	32	32	32	64	16	8
**C4**	16	8	32	32	8	16	4	4
**L5**	32	16	32	64	64	128	32	16
**C5**	32	16	32	64	16	16	8	8
**L6**	32	8	32	64	64	64	32	16
**C6**	32	8	64	64	16	8	8	8
**L7**	16	16	32	64	32	32	16	8
**C7**	16	16	128	128	16	16	8	8
L_mean_ *	20.6	13.7	32.0	54.9	73.1 ^†^	86.9 ^†^	25.1 ^†^	13.7 ^†^
C_mean_ *	13.7	10.9	59.4	77.7	16.0 ^†^	18.3 ^†^	7.7 ^†^	10.3 ^†^
*p*-value	0.201	0.338	0.085	0.307	0.003	0.003	0.004	0.097

* arithmetic mean of minimum biofilm inhibitory concentrations (MBIC) for linear (L) and cyclic (C) lipopeptides. † *p*-values for significant differences (*p* < 0.05).

**Table 5:** Hemolytic activity (HC50) and cytotoxicity (IC50) to HaCaT and HeLa.

Peptide Code	HC_50_ (μg/mL)	HaCaT IC_50_ (μg/mL)	HeLa IC_50_ (μg/mL)	SI _(IC50 HaCaT/ IC50 HeLa)_
**L1**	90.0 ± 6.5	5.2 ± 0.6	3.9 ± 1.1	1.3
**C1**	43.9 ± 3.5	23.6 ± 6.0	25.5 ± 2.9	0.9
**L2**	105.9 ± 8.0	23.5 ± 1.3	12.8 ± 1.1	1.8
**C2**	30.6 ± 1.1	26.9 ± 1.9	17.9 ± 2.1	1.5
**L3**	79.1 ± 6.1	23.6 ± 3.4	8.4 ± 1.7	2.8
**C3**	66.5 ± 14.8	36.8 ± 2.7	38.8 ± 4.4	0.9
**L4**	79.8 ± 9.0	4.3 ± 0.9	3.0 ± 0.6	1.4
**C4**	122.4 ± 16.5	33.8 ± 3.1	27.5 ± 3.4	1.2
**L5**	96.7 ± 1.4	2.4 ± 0.4	6.2 ± 2.7	0.4
**C5**	97.0 ± 15.4	12.9 ± 4.6	21.9 ± 4.5	0.6
**L6**	89.7 ± 2.4	7.5 ± 0.8	3.6 ± 0.5	2.1
**C6**	71.0 ± 6.4	45.7 ± 13.5	30.4 ± 2.5	1.5
**L7**	65.4 ± 1.5	8.1 ± 1.1	3.0 ± 0.4	2.7
**C7**	92.0 ± 9.6	21.3 ± 8.2	20.9 ± 2.1	1.0
L_mean_ *	86.7	10.7 ^†^	5.8 ^†^	1.8
C_mean_ *	74.7	28.7 ^†^	26.1 ^†^	1.1
*p*-value	0.375	0.013	0.00002	0.064

* arithmetic mean of HC_50_/IC_50_/SI for linear (L) and cyclic (C) lipopeptides. SI—selectivity indexes; ^†^ *p*-values for significant differences (*p* < 0.05).

**Table 6:** Analysis of selectivity indexes (left—linear, right—cyclic USCLs).

Bacteria		*S. aureus*		*S. epidermidis*		*E. coli*		*P. aeruginosa*	
hRBCs	Mean	6.17	8.52	13.60	13.51	8.61 ^†^	3.60 ^†^	2.41 ^†^	0.43 ^†^
	*p*-value	0.2964		0.9777		0.0039		0.0022	
HaCaT	Mean	1.37	2.04	1.61 ^†^	4.62 ^†^	0.97	1.26	0.39	0.23
	*p*-value	0.3452		0.0003		0.1600		0.7015	
**Fungi**		***C. albicans***		***C. glabrata***		***C. lipolytica***		***C. tropicalis***	
hRBCs	Mean	4.12 ^†^	19.59 ^†^	2.17 ^†^	15.77 ^†^	20.00	35.44	17.00	29.07
	*p*-value	0.0106		0.0022		0.0553		0.1599	
HaCaT	Mean	0.67 ^†^	8.36 ^†^	0.24 ^†^	6.29 ^†^	1.82 ^†^	13.39 ^†^	1.98 ^†^	12.29 ^†^
	*p*-value	0.0060		0.0022		0.0033		0.0049	

† *p*-values for significant differences (*p* < 0.05).

## Supplementary Materials

Supplementary materials can be found at https://www.mdpi.com/1422-0067/21/19/7208/s1. The following are available online, Figure S1: Cytotoxicity of linear (A) and cyclic (B) USCLs to HaCaT vs. adjusted retention time; Figure S2: Cytotoxicity of linear (A) and cyclic (B) USCLs to HeLa vs. adjusted retention time; Figure S3: Results of fluorescence measurements of membrane potential-sensitive probe *E. coli*; Figure S4: Results of fluorescence measurements of membrane potential-sensitive probe *S. aureus*; Figure S5: Snapshots from the POPG:POPE (Gram-positive bacterial membrane) binding simulations for C_16_-KKKK-NH_2_ (A) and C_16_-CKKKKC-NH_2_ (B); Figure S4: 2D density map of the lipopeptides and POPG lipids in the outer and inner leaflets of the POPG:POPE membrane (a grid spacing was set to 5 Å), local area per lipid (APL) and standard deviations of the local membrane APL and local thickness of the bilayer averaged over the last 100 ns of a total of 2 μs CG MD simulations of C_16_-KKKK-NH_2_ (A) and C_16_-CKKKKC-NH_2_ (B); Figure S7: Snapshots from the POPC:POPE:POPS:POPI:ERGO (fungal membrane) binding simulations for C_16_-KKKK-NH_2_ (A) and C_16_-CKKKKC-NH_2_ (B); Figure S8: 2D density map of the lipopeptides and POPI lipids in the outer and inner leaflets of the POPC:POPE:POPS:POPI:ERGO membrane (a grid spacing was set to 5 Å), local area per lipid (APL) and standard deviations of the local membrane APL and local thickness of the bilayer averaged over the last 100 ns of a total of 2 μs CG MD simulations of C_16_-KKKK-NH_2_ (A) and C_16_-CKKKKC-NH_2_ (B); Figure S9: Snapshots from the POPG:POPE (Gram-positive bacterial membrane) binding simulations for C_16_-KRKK-NH_2_ (A) and C_16_-CKRKKC-NH_2_ (B); Figure S10: 2D density map of the lipopeptides and POPG lipids in the outer and inner leaflets of the POPG:POPE membrane (a grid spacing was set to 5 Å), local area per lipid (APL) and standard deviations of the local membrane APL and local thickness of the bilayer averaged over the last 100 ns of a total of 2 μs CG MD simulations of C_16_-KRKK-NH_2_ (A) and C_16_-CKRKKC-NH_2_ (B); Table S1: The SI of the tested compounds.

## Abbreviations

BSA	Bovine serum albumin
CENTA	7-(thienyl-2-acetamido)-3-[(4-nitro-3-carboxyphenyl)thiomethyl]-3-cephem-4 carboxylic acid
CFU	Colony forming units
DCM	Dichloromethane
DIC	*N,N’*-diisopropylcarbodiimide
DiSC3(5)	3,3′-dipropylthiacarbocyanine iodide
DMF	*N,N*-dimethylformamide
DMSO	Dimethyl sulfoxide
EDT	1,2-ethaneditiol
EDTA	Ethylenediaminetetraacetic acid
ESI-MS	Electrospray ionization mass spectrometry
FM 1-43	*N-*(3-triethylammoniumpropyl)-4-(4-(dibutylamino) styryl) pyridinium dibromide
Fmoc	Fluorenylmethoxycarbonyl
HaCaT	Human keratinocytes
HC50	Peptide concentration causing 50% hemolysis
HeLa	Human cervical adenocarcinoma cell line
hRBCs	Human red blood cells
IC50	Peptide concentration causing 50% inhibition of the growth
IM	Inner membrane
LB	Luria-Bertani medium
MBEC	Minimum biofilm eradication concentration
MBIC	Minimum biofilm inhibitory concentration
MHA	Mueller-Hinton agar
MHB	Mueller-Hinton broth
MIC	Minimum inhibitory concentration
MTT	3-(4,5-dimethylthiazol-2-yl)-2,5-diphenyltetrazolium bromide
ONPG	o-nitrophenyl-β-d-galactopyranoside
OM	Outer membrane
Pal	Palmitic acid (hexadecenoic acid)
PI	Propidium iodide
POPC	1-palmitoyl-2-oleoyl-sn-glycero-3-phosphocholine
POPE	1-palmitoyl-2-oleoyl-sn-glycero-3-phosphoethanolamine
POPG	1-palmitoyl-2-oleoyl-sn-glycero-3-phosphoglycerol
POPI	1-palmitoyl-2-oleoyl-sn-glycero-3-phosphoinositol
POPS	1-palmitoyl-2-oleoyl-sn-glycero-3-phosphoserine
RP-HPLC	reversed-phase high-performance liquid chromatography
SDA	Sabouraud dextrose agar
SDB	Sabouraud dextrose broth
SI	Selectivity index
tBu	*tert*-butyl
TFA	Trifluoroacetic acid
TIS	Triisopropylsilane
