# Supplementary material for: Effect of Disulfide Cyclization of Ultrashort Cationic Lipopeptides on Antimicrobial Activity and Cytotoxicity
Source: Int J Mol Sci. 2020 Sep 29;21(19):7208. doi: 10.3390/ijms21197208 (PMC7582905; doi:10.3390/ijms21197208)
Supplement: Supplementary file 1 [file ijms-21-07208-s001.pdf]

International Journal of *Molecular Sciences*

MDPI

Supplementary Materials

Table S1. The SI of the tested compounds. SI = HC50/MIC or IC50/MIC.

Peptide Code	Selectivity Indices (SI) Concerns MICs, HC50 (Left) and $IC50_{HaCaT}$ (Right)															
	S. aureus		*S. epidermidis*		*E. coli*		*P. aeruginosa*		*C. albicans*		*C. glabrata*		*C. lipolytica*		*C. tropicalis*	
L1	2.81	0.16	11.25	0.65	5.63	0.33	1.41	0.08	2.81	0.16	2.81	0.16	5.63	0.33	11.25	0.65
C1	6.62	1.47	10.98	5.90	2.74	1.48	0.17	0.09	5.49	2.95	10.98	5.90	10.98	5.90	10.98	5.90
L2	4.94	1.48	13.24	2.94	3.31	0.73	6.62	1.47	3.31	0.73	1.65	0.37	13.24	2.94	26.48	5.88
C2	9.98	0.54	3.83	3.36	0.48	0.42	0.96	0.84	7.65	6.73	7.65	6.73	15.30	13.45	15.30	13.45
L3	6.04	0.15	9.89	2.95	9.89	2.95	2.47	0.74	9.89	2.95	1.24	0.37	9.89	2.95	9.89	2.95
C3	5.61	0.47	8.31	4.60	4.16	2.30	0.13	0.07	33.25	18.40	8.31	4.60	16.63	9.20	33.25	18.40
L4	16.35	2.03	9.98	0.54	9.98	0.54	2.49	0.13	4.99	0.27	2.49	0.13	79.80	4.30	39.90	2.15
C4	5.49	2.95	15.30	4.23	3.83	1.06	0.48	0.13	61.20	16.90	30.60	8.45	122.40	33.80	61.20	16.90
L5	0.96	0.84	12.09	0.30	12.09	0.30	1.51	0.04	3.02	0.08	1.51	0.04	12.09	0.30	12.09	0.30
C5	8.31	4.60	24.25	3.23	6.06	0.81	0.38	0.05	6.06	0.81	12.13	1.61	24.25	3.23	24.25	3.23
L6	7.65	2.11	22.43	1.88	11.21	0.94	1.40	0.12	2.80	0.23	1.40	0.12	11.21	0.94	11.21	0.94
C6	12.13	1.61	8.88	5.71	2.22	1.43	0.55	0.36	17.75	11.43	17.75	11.43	35.50	22.85	35.50	22.85
L7	4.44	2.86	16.35	2.03	8.18	1.01	1.02	0.13	2.04	0.25	4.09	0.51	8.18	1.01	8.18	1.01
C7	11.50	2.66	23.00	5.33	5.75	1.33	0.36	0.08	5.75	1.33	23.00	5.33	23.00	5.33	23.00	5.33

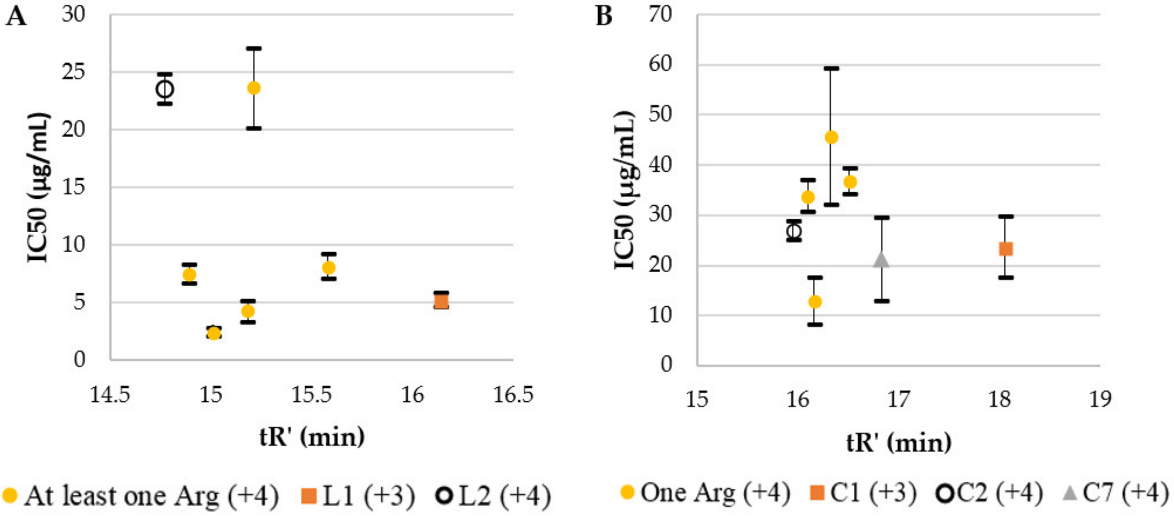

Figure S1. Cytotoxicity of linear (**A**) and cyclic (**B**) USCLs to HaCaT vs adjusted retention time. (**A**) At least one Arg (compounds **L3**, **L4**, **L5**, **L6**, **L7**); (**B**) One Arg (compounds **C3**, **C4**, **C5**, **C6**).

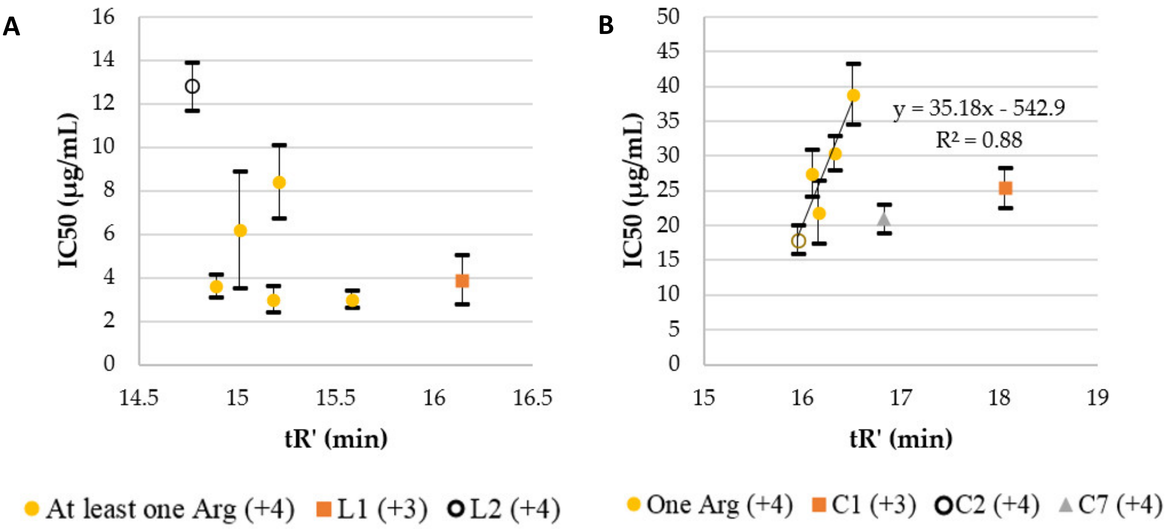

Figure S2. Cytotoxicity of linear (**A**) and cyclic (**B**) USCLs to HeLa vs adjusted retention time. (**A**) At least one Arg (compounds **L3**, **L4**, **L5**, **L6**, **L7**); (**B**) One Arg (compounds **C3**, **C4**, **C5**, **C6**).

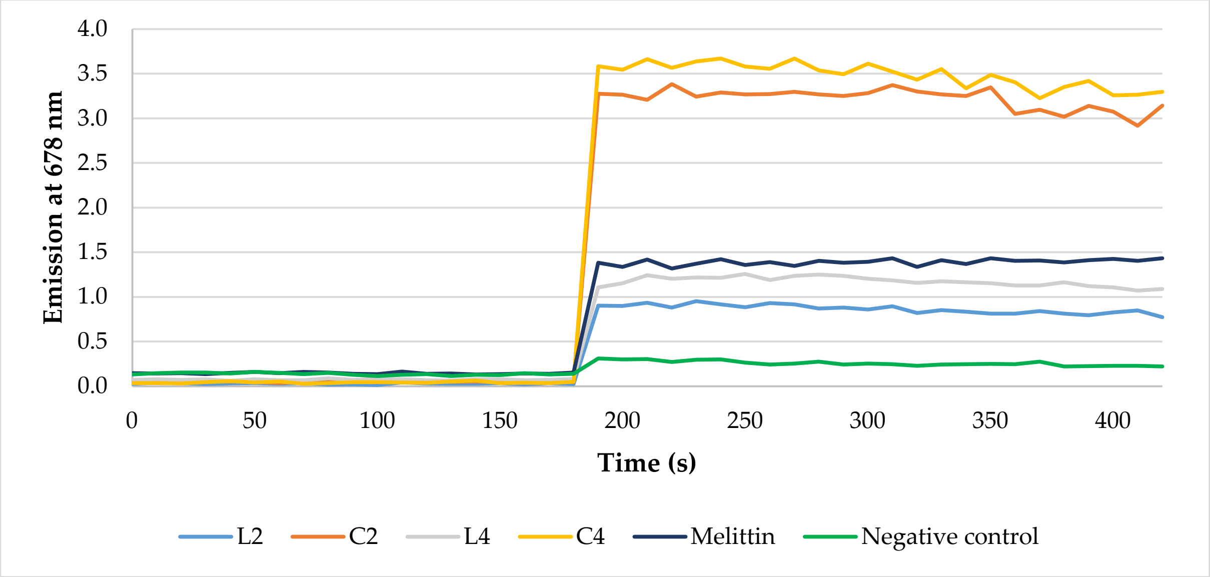

Figure S3. Results of fluorescence measurements of membrane potential-sensitive probe – *E. coli*.

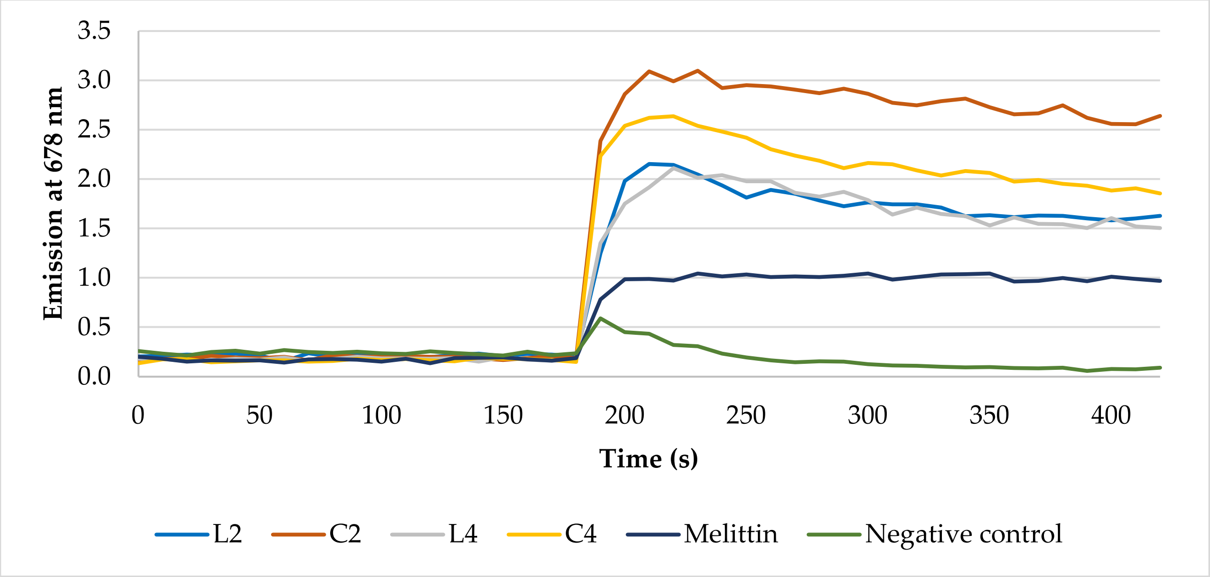

Figure S4. Results of fluorescence measurements of membrane potential-sensitive probe – *S. aureus*.

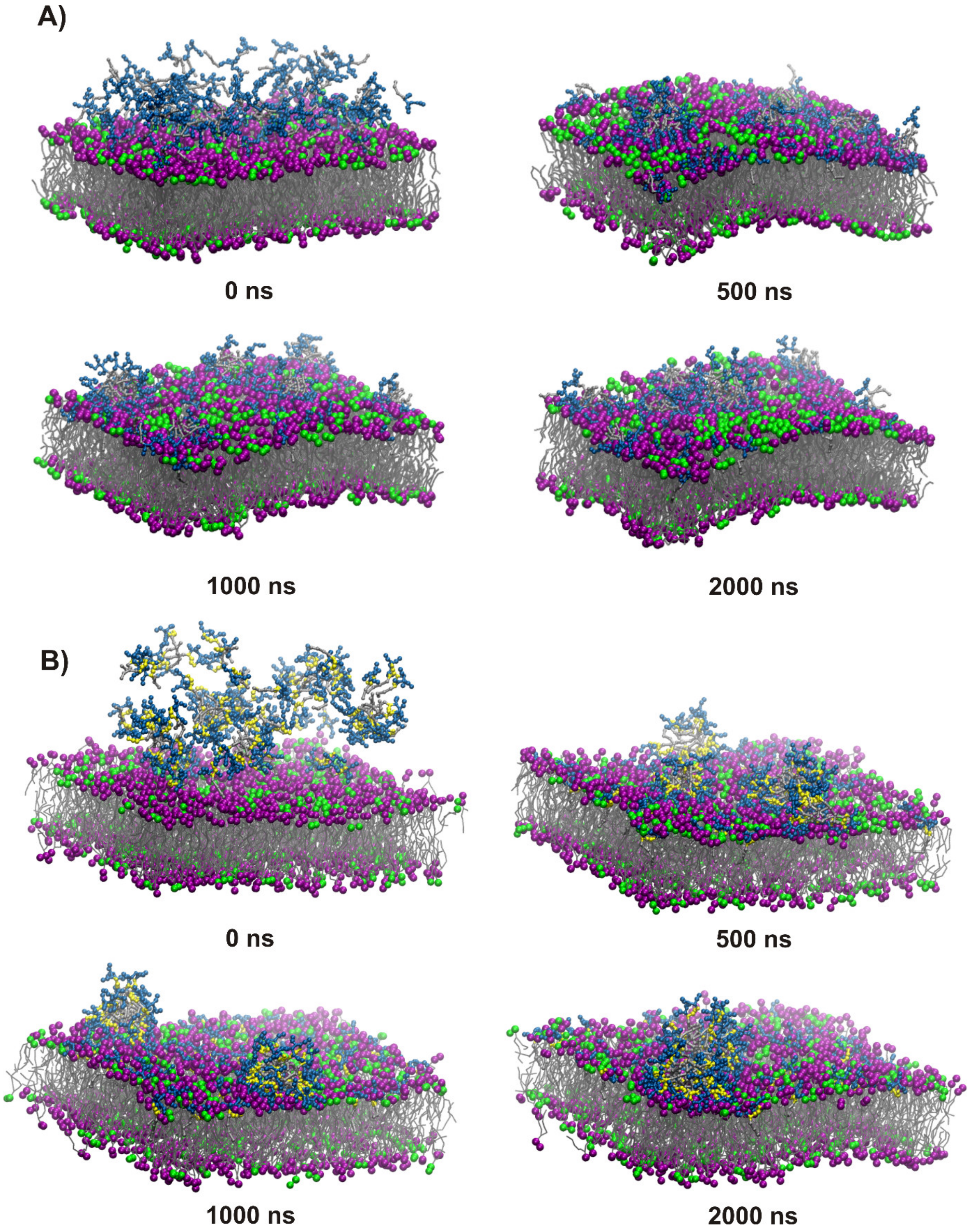

Figure S5. Snapshots from the POPG:POPE (Gram-positive bacterial membrane) binding simulations for C_{16}-KKKK-NH_2 (**A**) and C_{16}-CKKKKC-NH_2 (**B**).

Fatty acid tails are colored silver, while lysines, arginines and cysteines are blue, cyan and yellow, respectively. Lipid tails are gray, while lipid headgroups are purple for POPG and green for POPE. In case of C_{16}-KKKK-NH_2, the MD simulations previously published were used for the analysis [1].

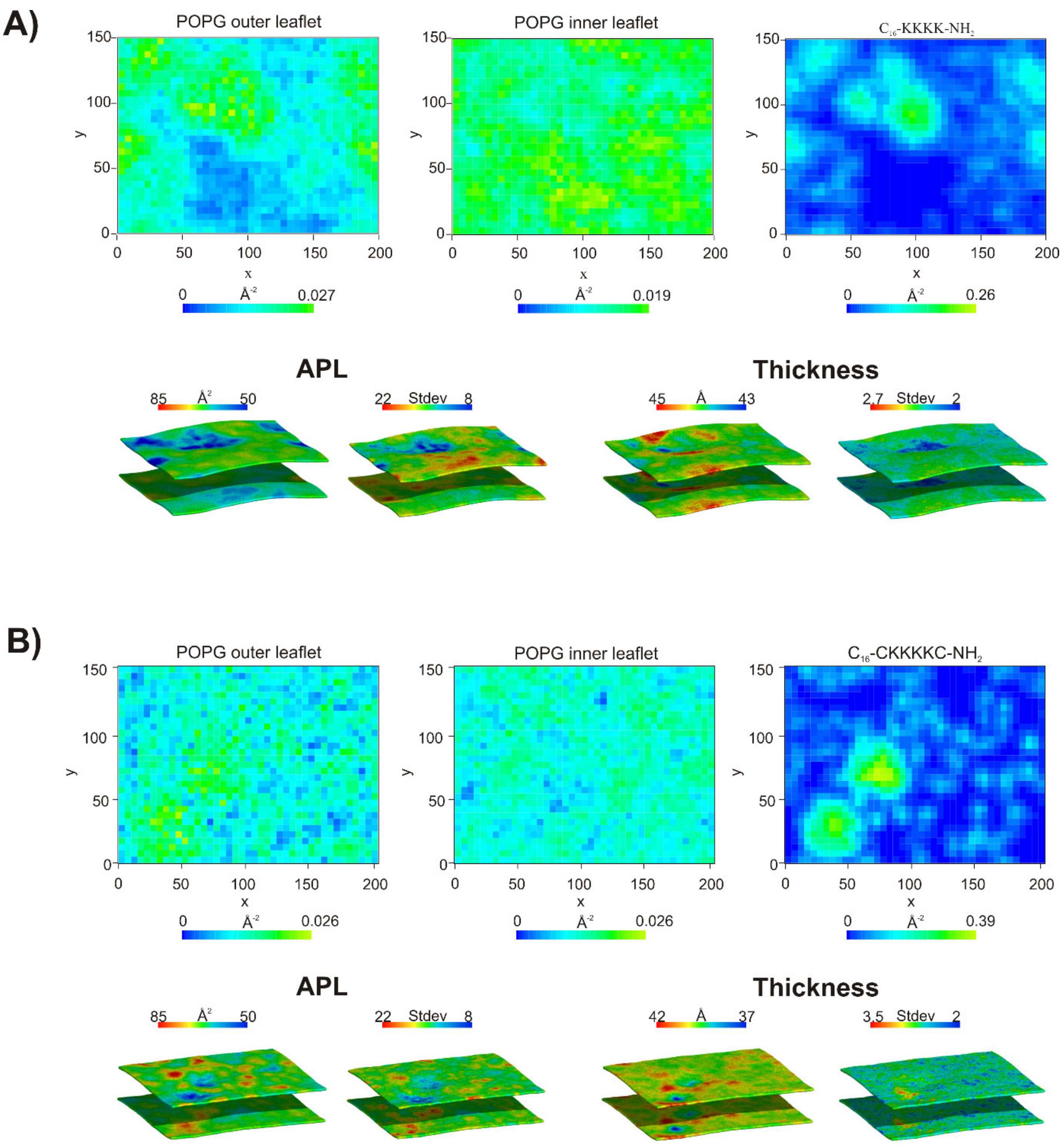

Figure S6. 2D density map of the lipopeptides and POPG lipids in the outer and inner leaflets of the POPG:POPE membrane (a grid spacing was set to 5 Å), local area per lipid (APL) and standard deviations of the local membrane APL and local thickness of the bilayer averaged over the last 100 ns of a total of 2 μs CG MD simulations of C_{16}-KKKK-NH_2 (**A**) and C_{16}-CKKKKC-NH_2 **(B)**.

Phosphate beads of the lipid headgroups were considered for calculations. In case of C_{16}-KKKK-NH_2, the MD simulations previously published were used for the analysis [1]. Note different scale for membrane thickness in case of C_{16}-KKKK-NH_2.

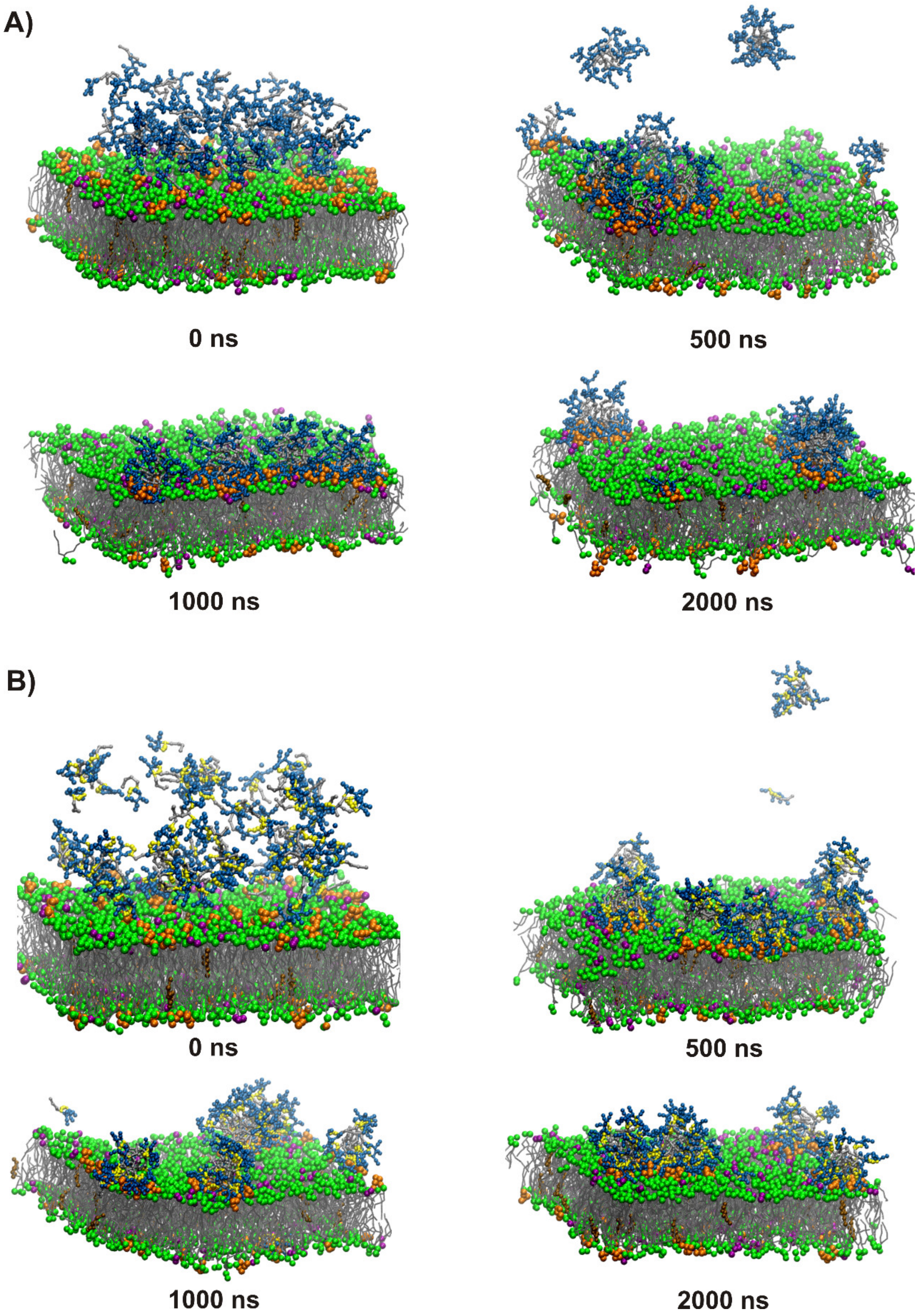

Figure S7. Snapshots from the POPC:POPE:POPS:POP1:ERGO (fungal membrane) binding simulations for C_{16}-KKKK-NH_2 (**A**) and C_{16}-CKKKKC-NH_2 (**B**).

Fatty acid tails are colored silver, while lysines, arginines and cysteines are blue, cyan and yellow, respectively. Lipid tails are gray, while lipid head groups are purple for POPG, green for POPC and POPE, and orange for POP1. Ergosterol (brown) is immersed in hydrophobic part of the membrane.

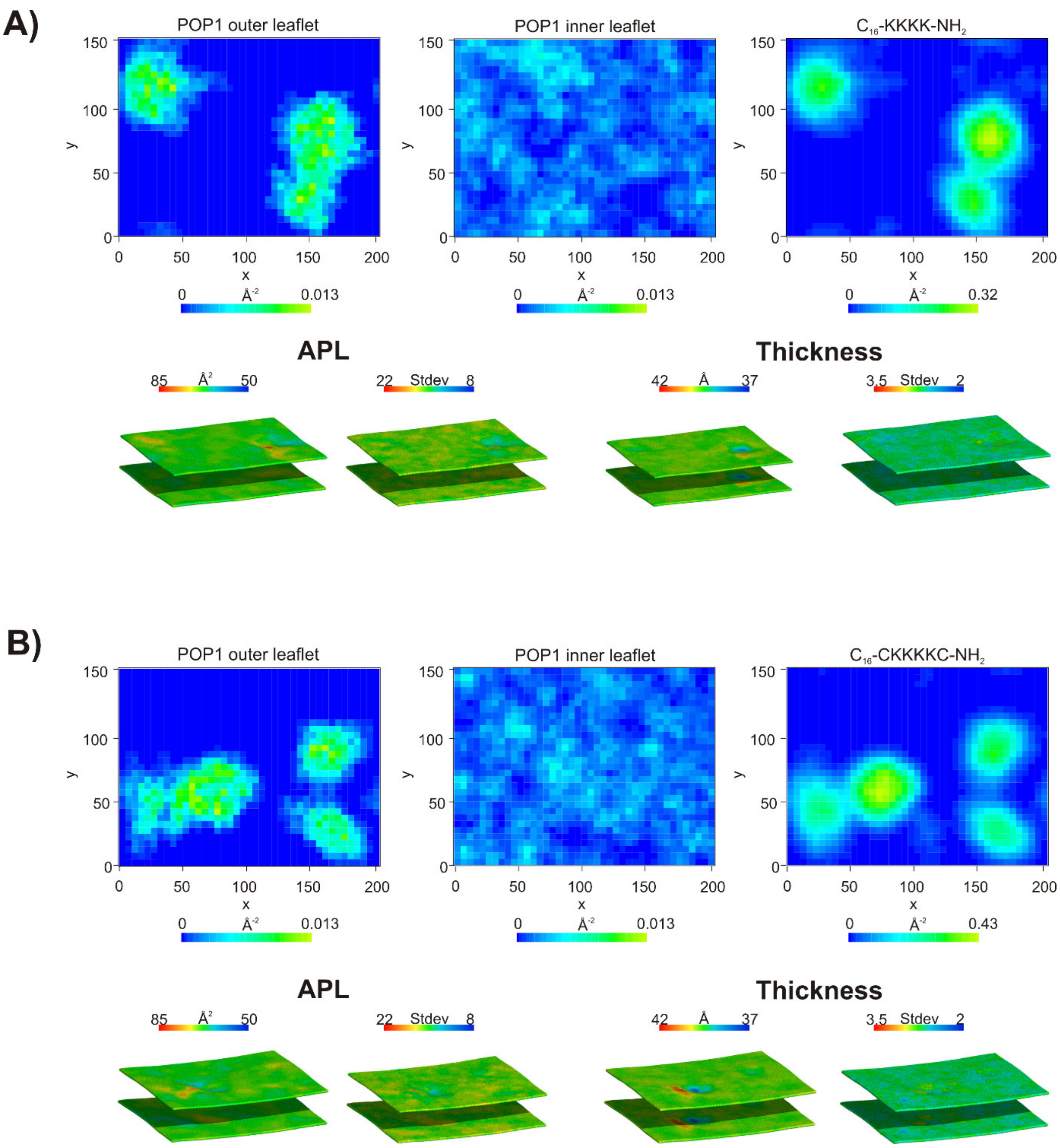

Figure S8. 2D density map of the lipopeptides and POP1 lipids in the outer and inner leaflets of the POPC:POPE:POPS:POP1:ERGO membrane (a grid spacing was set to 5 Å), local area per lipid (APL) and standard deviations of the local membrane APL and local thickness of the bilayer averaged over the last 100 ns of a total of 2 µs CG MD simulations of C_{16}-KKKK-NH_2 (**A**) and C_{16}-CKKKKC-NH_2 **(B)**. Phosphate beads of the lipid headgroups were considered for calculations.

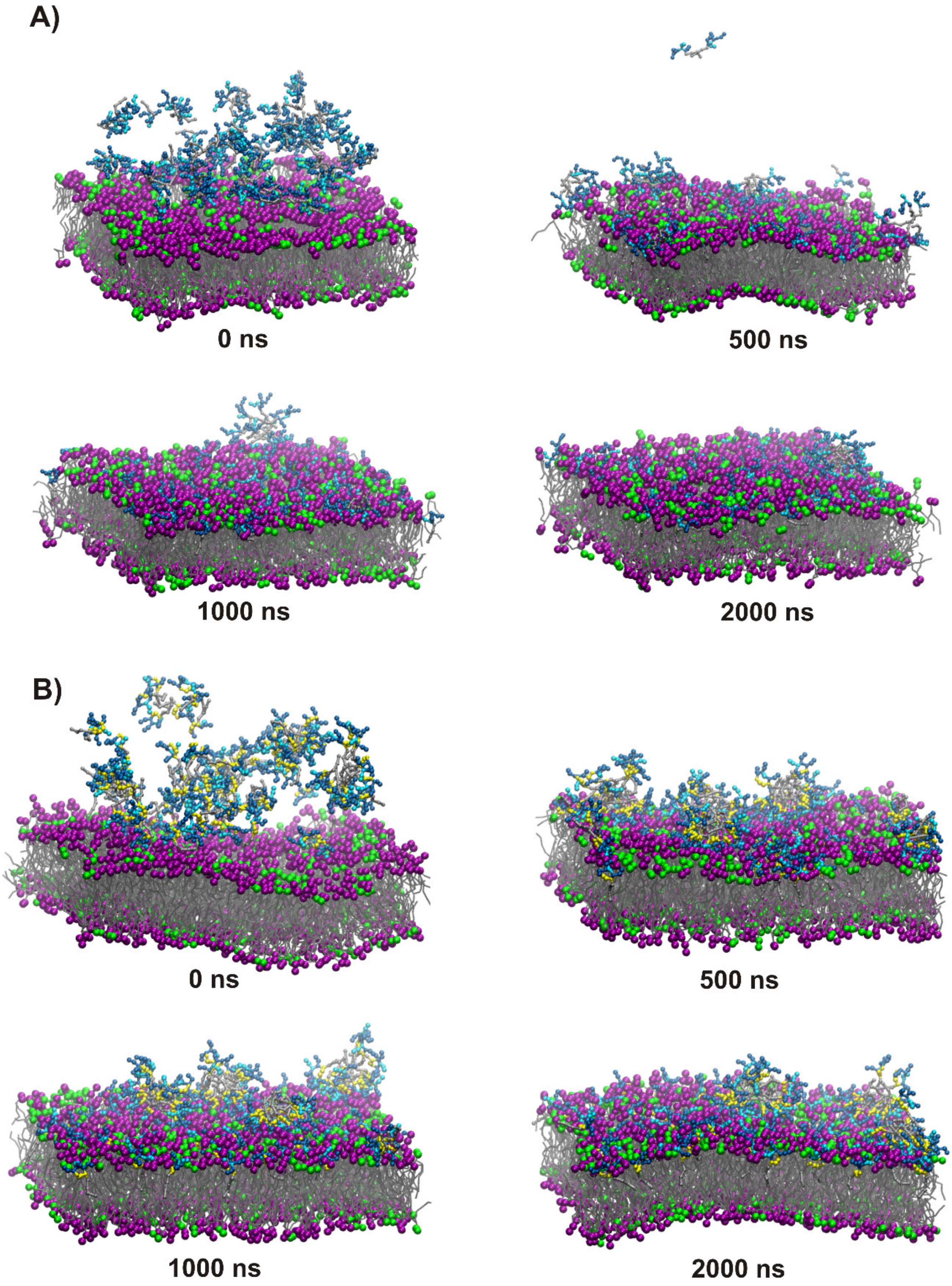

Figure S9. Snapshots from the POPG:POPE (Gram-positive bacterial membrane) binding simulations for C_{16}-KRKK-NH_2 (**A**) and C_{16}-CKRKKC-NH_2 (**B**).

Fatty acid tails are silver, while lysines, arginines and cysteines are blue, cyan and yellow, respectively. Lipid tails are gray, while lipid headgroups are purple for POPG and green for POPE.

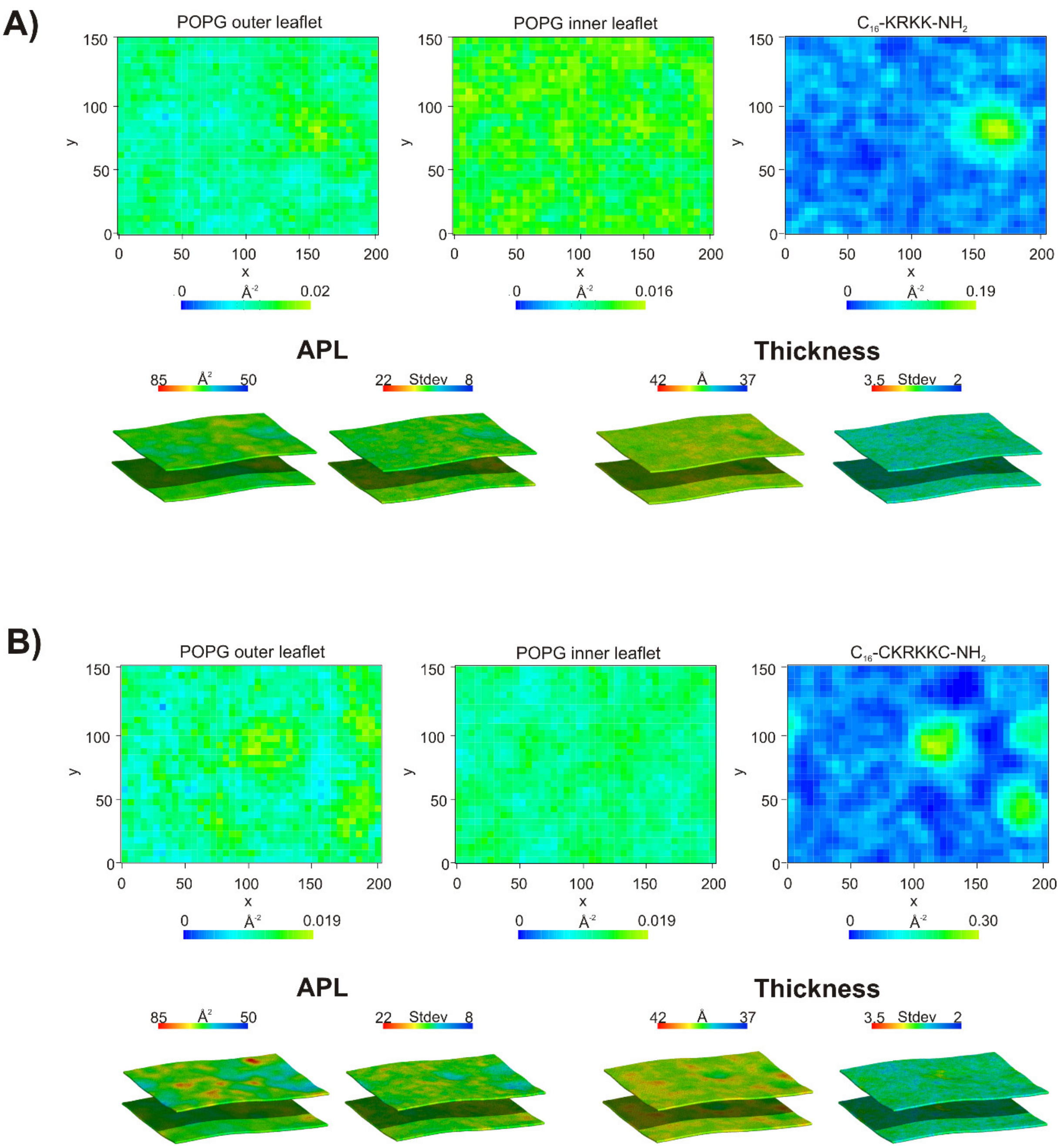

Figure S10. 2D density map of the lipopeptides and POPG lipids in the outer and inner leaflets of the POPG:POPE membrane (a grid spacing was set to 5 Å), local area per lipid (APL) and standard deviations of the local membrane APL and local thickness of the bilayer averaged over the last 100 ns of a total of 2 μs CG MD simulations of C_{16}-KRKK-NH_2 (**A**) and C_{16}-CKRKKC-NH_2 **(B)**. Phosphate beads of the lipid headgroups were considered for calculations.

Reference:

Sikorska, E.; Dawgul, M.; Greber, K.; Iłowska, E.; Pogorzelska, A.; Kamysz, W. Self-assembly and interactions of short antimicrobial cationic lipopeptides with membrane lipids: ITC, FTIR and molecular dynamics studies. *Biochim. Biophys. Acta* **2014**, *1838*, 2625–2634, doi:10.1016/j.bbamem.2014.06.016.

© 2020 by the author. Licensee MDPI, Basel, Switzerland. This article is an open access article distributed under the terms and conditions of the Creative Commons Attribution (CC BY) license (http://creativecommons.org/licenses/by/4.0/).
